# Amino acid levels determine metabolism and CYP450 function of hepatocytes and hepatoma cell lines

**DOI:** 10.1038/s41467-020-15058-6

**Published:** 2020-03-13

**Authors:** Ruben Boon, Manoj Kumar, Tine Tricot, Ilaria Elia, Laura Ordovas, Frank Jacobs, Jennifer One, Jonathan De Smedt, Guy Eelen, Matthew Bird, Philip Roelandt, Ginevra Doglioni, Kim Vriens, Matteo Rossi, Marta Aguirre Vazquez, Thomas Vanwelden, François Chesnais, Adil El Taghdouini, Mustapha Najimi, Etienne Sokal, David Cassiman, Jan Snoeys, Mario Monshouwer, Wei-Shou Hu, Christian Lange, Peter Carmeliet, Sarah-Maria Fendt, Catherine M. Verfaillie

**Affiliations:** 1 Department of Development and Regeneration, Stem Cell Institute, KU Leuven, Leuven, Belgium; 2 Laboratory of Cellular Metabolism and Metabolic Regulation, VIB Center for Cancer Biology, VIB, Leuven, Belgium; 3 Laboratory of Cellular Metabolism and Metabolic Regulation, Department of Oncology, KU Leuven and Leuven Cancer Institute (LKI), Leuven, Belgium; 4 Janssen Research and Development, Beerse, Belgium; 5 Department of Chemical Engineering and Materials Science, University of Minnesota, Minneapolis, MN USA; 6 Stem Cell Institute, University of Minnesota, Minneapolis, MN USA; 7 Laboratory of Angiogenesis and Vascular Metabolism, Department of Oncology, KU Leuven, Leuven, Belgium; 8 Laboratory of Angiogenesis and Vascular Metabolism, Center of Cancer Biology, VIB, Leuven, Belgium; 9 Hepatology, Department of Clinical and Experimental Medicine, KU Leuven, Leuven, Belgium; 10 Laboratory of Pediatric Hepatology and Cell Therapy, Universit Catholique de Louvain & Cliniques Universitaires St Luc, Institut de Recherche Clinique et Expérimentale (IREC), Brussels, Belgium; 11 Present Address: Biomedical Signal Interpretation and Computational Simulation (BSICoS) Group, Aragón Institute of Engineering Research, IIS Aragón University of Zaragoza, Aragon I + D Foundation (ARAID), Zaragoza, Spain; 12 Present Address: Translational Research in GastroIntestinal Disorders (TARGID), Department of Chronic Diseases, Metabolism and Ageing (CHROMETA), KU Leuven, Leuven, Belgium; 13 Present Address: Department of Gastroenterology and Hepatology, UZ Leuven, Leuven, Belgium

**Keywords:** Metabolic engineering, Metabolic pathways, High-throughput screening, Stem-cell differentiation

## Abstract

Predicting drug-induced liver injury in a preclinical setting remains challenging, as cultured primary human hepatocytes (PHHs), pluripotent stem cell-derived hepatocyte-like cells (HLCs), and hepatoma cells exhibit poor drug biotransformation capacity. We here demonstrate that hepatic functionality depends more on cellular metabolism and extracellular nutrients than on developmental regulators. Specifically, we demonstrate that increasing extracellular amino acids beyond the nutritional need of HLCs and HepG2 cells induces glucose independence, mitochondrial function, and the acquisition of a transcriptional profile that is closer to PHHs. Moreover, we show that these high levels of amino acids are sufficient to drive HLC and HepG2 drug biotransformation and liver-toxin sensitivity to levels similar to those in PHHs. In conclusion, we provide data indicating that extracellular nutrient levels represent a major determinant of cellular maturity and can be utilized to guide stem cell differentiation to the hepatic lineage.

## Abstract (web summary)

Hepatocytes grown in a dish are immature and do not metabolize compounds as a real liver would. Here, the authors supply stem cell-derived hepatocytes with amino acids at a higher concentration than nutritionally necessary, changing the metabolism of these cells, making them more mature and useful for drug screening and toxicity studies.

## Introduction

Despite robust preclinical rodent studies, unpredicted liver toxicity remains one of the major reasons for drug failure^1^. Hepatocytes express a set of highly specific cytochrome P450 (CYP450) biotransforming enzymes that convert many drugs to inactive or hepatotoxic compounds. As these CYP450 enzymes differ significantly between species, drug biotransformation and hepatotoxicity can only be evaluated in freshly isolated primary human hepatocytes (PHHs)^2^. However, differences between donors necessitate the screening of multiple PHH batches and rapid loss of hepatic function in two-dimensional (2D) culture does not allow for PHH expansion nor long-term drug exposure studies^3,4^. As a result, novel systems are being developed to better maintain PHH function for an extended time^5–7^. Alternative assays utilize hepatic cell lines such as the HepG2 (ref. ^8^) and HepaRG models^9^, or transiently immortalized PHHs^10^. Nevertheless, as these models still do not reach drug biotransformation capacities of fresh PHHs, the major goal of toxicologists remains the generation of expandable models with stable CYP450 activity levels close to those of fresh PHH^11,12^.

Hepatocytes derived from pluripotent stem cells (PSCs) represent a highly versatile model^13^. PSCs are genetically normal and can be expanded almost indefinitely. In addition, induced PSC (iPSC) technology allows for the generation of models from individuals with different susceptibility profiles^14^. PSCs have been differentiated to cells that secrete albumin, store glycogen, and are susceptible to infection with hepatotropic viruses^15,16^. However, these cells are not mature and have therefore been termed “hepatocyte-like cells” (HLCs). HLCs differ from PHHs at the transcriptional, proteomic, and epigenetical level, and specifically express low levels of hepatic transcription factors (TFs) and CYP450 enzymes^4,17,18^. Recent studies have shown that utilizing signals from the in vivo microenvironment can improve HLC maturation. These approaches include co-culture with non-parenchymal liver cells, micro-patterning on fibroblasts, and adapting the culture system to three-dimensional-hydrogel or even perfused systems, often combined with overexpression of TFs to further boost functionality. Nevertheless, HLCs still cannot predict hepatotoxicity at clinically relevant drug concentrations^19–23^.

In recent years, cellular metabolism has emerged as an important regulator of PSC fate^24,25^. Yet, even if single metabolites were found to drive PSC differentiation to the neural lineage^26,27^, the impact of nutrients on hepatic differentiation and function remains unknown^28^. We here demonstrate that the immature profile of HLCs is in fact determined by an immature metabolic profile. We also show that extracellular amino acids (AAs) represent a tool to drive both metabolic maturation and the expression of CYP450 enzymes, and this in both HLCs and HepG2 hepatoma cells. Finally, by adapting the model to a high-throughput toxicity screening system, we demonstrate that AAs drive hepatic differentiation to levels that allow detection of hepatotoxins at clinically relevant concentrations.

## Results

### PSC differentiation yields immature hepatic progeny

**Fig. 1:**
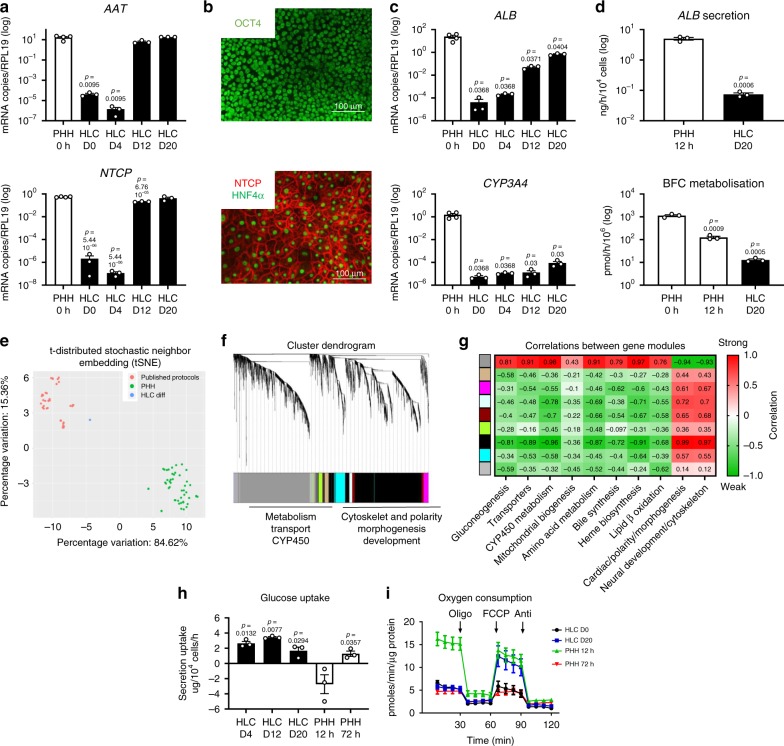
HLCs and PHHs cultured in 2D are functionally and metabolically immature. **a** Expression of *AAT* and *NTCP* in PHHs and differentiating HLCs. *N* = 3 independent differentiations, *N* = 4 donors for PHHs. Significance was compared with PHHs by unpaired two-tailed Student’s *t*-test. **b** Representative immunofluorescence images of two independent differentiations with OCT4 staining on D0 and HNF4A/NTCP staining on D20. Scale bar represents 100 µm. **c** Expression of *ALB* and *CYP3A4* in PHHs and differentiating HLCs. *N* = 3 independent differentiations, *N* = 4 donors for PHHs. Significance was compared with PHHs by unpaired two-tailed Student’s *t*-test. **d** Functional comparison between HLC D20 and PHH 12 h for albumin secretion and BFC metabolisation. *N* = 3 independent differentiations, *N* = 3 donors for suspension PHH 0 h and plated PHH 12 h. Significance was compared with PHH 0 h (BFC metabolisation) or with PHH 12 h (Albumin secretion) by unpaired two-tailed Student’s *t*-test. **e** t-SNE plot representing HLCs generated by different protocols and PHHs. **f** WGCNA analysis for genes differentially expressed between HLCs to PHHs. **g** Representation of correlation of molecular pathways to different modules. Correlation was shown as strong (red color) or weak (green color). **h** Glucose uptake/secretion over 24 h of culture in differentiating HLCs and cultured PHHs, *N* = 3 independent differentiations, *N* = 3 donors for PHHs. Significance was compared with PHH 12 h by unpaired two-tailed Student’s *t*-test. **i** Oxygen consumption rates with addition of oligomycin, FCCP, and antimycin for HLCs (D0 and D20), PHH 12 h, and PHH-72h. *N* = 6 wells, 5 time point measurements per well. For PHHs, *N* = 6 wells for each of 2 donors, 5 time point measurements per well. Data in all panels represent mean ± SEM with *P*-values indicated when significant. Source data are provided as a Source Data file.

To evaluate and improve HLC function, we first compared hepatic progeny of the human H9 embryonic stem cell line to cryopreserved PHHs. As expected, during differentiation from PSC, expression of pluripotency-associated genes decreased from day 0 onwards, whereas endoderm-associated genes were transiently induced (Supplementary Fig. 1A). By day 20, hepatic transcripts, such as alpha1-antitrypsin (*AAT)* and Na^+^ -taurocholate cotransporting polypeptide (*NTCP)*, reached levels found in PHHs (Fig. 1a). Immunostaining and flow cytometry demonstrated that more than 90% of HLCs stained positive for NTCP, hepatocyte nuclear factor (HNF)4A and AAT (Fig. 1b and Supplementary Fig. 1B). However, in line with other protocols^17^, expression (Fig. 1c) and activity (Fig. 1d) of mature genes such as albumin (*ALB*) or *CYP3A4*, the most important drug-biotransforming enzyme, remained lower than in PHHs or even 12 h plated PHHs (PHH 12 h). Consistently, the transcriptome of generated HLCs clustered together with published protocols, at a distance of PHHs (Fig. 1e). To gain molecular insights into the low *CYP450* expression of HLCs, we used a weighted correlation network analysis (WGCNA)^29^ to identify two gene clusters of transcriptional regulators that differ between HLCs and PHHs. As previously described^4,17^, one of these contained genes involved in the development and cytoskeleton. Surprisingly, *CYP450* genes were found to be co-regulated with metabolic rather than developmental genes (Fig. 1f). When we assessed the trait relation between the different clusters (Fig. 1g), we found a strong correlation between modules containing *CYP450* genes and genes involved in gluconeogenesis, mitochondrial metabolism, AA metabolism, and β-oxidation. As a lower correlation was found with modules linked to development, polarity, and cytoskeleton, we hypothesized that an immature metabolism is the prime reason for low *CYP450* expression.

PSCs are glycolytic whereas hepatocytes represent the main gluconeogenic cell type. However, during hepatic differentiation, transcript levels of the glycolytic genes hexokinase II (*HKII)* and pyruvate kinase (*PK)M2* remained high, whereas the gluconeogenic genes (glucose 6 phosphatase (*G6PC*), fructose-bisphosphatase 1 (*FBP1*), phosphoenolpyruvate carboxykinase (*PEPCK*)) and the liver-specific PKL isoform were not induced (Supplementary Fig. 1C). This glycolytic phenotype was confirmed by a continued high rate of glucose uptake (Fig. 1h). In accordance, measurements of oxygen consumption rates (OCRs) confirmed a very low basal mitochondrial activity, but not maximal respiratory capacity (measured through addition of carbonyl cyanide-4-(trifluoromethoxy) phenylhydrazone (FCCP)) when comparing HLC D20 with PHH 12 h (Fig. 1i). As also demonstrated by others^7^, PHHs quickly lose their mature features such *ALB* and *CYP3A4* expression upon culturing (Supplementary Fig. 1D, E). Interestingly, we also observed a switch from a gluconeogenic to a glycolytic gene expression profile (Supplementary Fig. 1E), a switch from glucose secretion to consumption (Fig. 1h), and reduction in basal OCR and maximal reserve capacity in PHHs cultured for 72 h (PHH 72 h) (Fig. 1i). This demonstrates that dedifferentiating PHHs and HLCs display an immature metabolism and minimal expression of drug-biotransforming genes.

### Transcription factors regulate hepatic metabolism and function

The RNA-sequencing (RNAseq) studies, confirmed by quantitative reverse-transcription PCR (qRT-PCR) (Supplementary Fig. 2A), also identified a number of hepatic TFs to be less expressed in HLC D20 compared with PHHs. As overexpression of hepatic TFs has been shown to enhance CYP450 activity to some extent^23,30^, we next assessed whether these might also rewire hepatic metabolism. We therefore utilized recombinase-mediated cassette exchange (RMCE)^31^ to generate PSCs containing a doxycycline-inducible cassette for the overexpression of *HNF1A*, forkhead box *(FOX)A3* and Prospero homeobox protein (*PROX1*) (termed HC3X). Correct integration of all cassettes was validated using PCR and fluorescence-activated cell sorting (Supplementary Fig. 2B, C). As a control, we recombined tandem dimer tomato (tdT) and demonstrated that ±100% of the cells were tdT positive following doxycycline addition (Supplementary Fig. 2D).

**Fig. 2:**
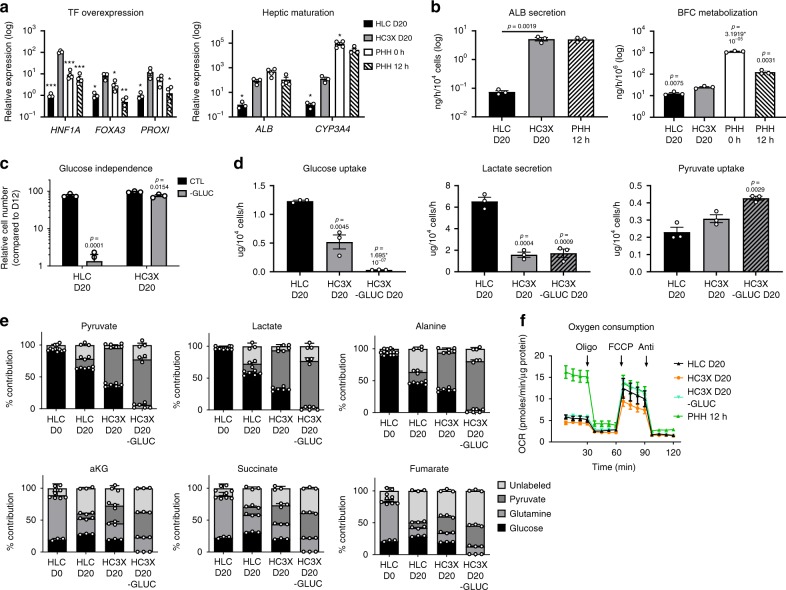
Overexpression of *HNF1A*, *FOXA3*, and *PROX1* induces partial functional and metabolic maturation. **a** Relative gene expression analysis. *N* = 3 independent differentiations for HLC and HC3X, *N* = 4 donors for PHH. Significance was calculated compared with HC3X D20 by unpaired two-tailed Student’s *t*-test. *p < 0.05, ***p < 0.001. **b** Functional comparison between HLC D20, HC3X D20, and PHH 12 h. Data for HLC and PHH 12 h are repeated from Fig. 1d. *N* = 3 independent differentiations for HLC and HC3X; *N* = 3 donors for plated PHH 12 h. Significance was calculated compared with HC3X D20 unpaired two-tailed Student’s *t*-test. **c** Relative cell number (%) comparing CTL media and media without glucose (−Glucose) for HLC D20 and HC3X D20. *N* = 3 independent differentiations. Significance was calculated by comparing differentiation with and without glucose by unpaired two-tailed Student’s *t*-test. **d** Glucose, lactate, and pyruvate uptake or secretion in HLC D20 and HC3X D20, the latter grown with (HC3X) or without (HC3X-GLUC) glucose. *N* = 3 independent differentiations; *N* = 3 donors for PHHs. Significance was calculated by comparing differentiations with HLC D20 by unpaired two-tailed Student’s *t*-test. **e** Percentage of ^13^C-labeled glucose, glutamine, or pyruvate contribution to pyruvate, lactate, alanine, alpha-ketoglutarate (aKG), succinate, and fumarate. The non-labeled fraction was designated as unlabeled. *N* = 3 independent differentiations. **f** Oxygen consumption rates with addition of oligomycin, FCCP, and antimycin for HC3X D20 cultured with glucose (HC3X D20) or without glucose (HC3X D20 –GLUC). Data for HLC D20 and PHH 12 h are repeated from Fig. 1g. *N* = 6 wells, 5 time point measurements per well. PHH = *N* = 6 wells for 2 different donors, 5 time point measurements per well. Data in all panels represent mean ± SEM with *P*-values indicated when significant. Source data are provided as a Source Data file.

Induction of *HNF1A*, *FOXA3*, and *PROX1* from D4 until D20 induced their expression to levels near those of PHHs (Fig. 2a) and increased both *ALB* and *CYP3A4* mRNA. Transcript levels of *ALB* now reached those of PHH 12 h and *CYP3A4* expression was increased 50-fold (Fig. 2a). Although albumin secretion by HC3X D20 was found to be equal to PHH 12 h, the metabolization rate of the probe compound 7-benzyloxy-4-trifluoromethylcoumarin (BFC) was still very low (Fig. 2b). Overexpression was associated with partial metabolic maturation. Transcripts for glycolytic enzymes were decreased in HC3X D20, whereas expression of *G6PC* and *PEPCK* were modestly increased (Supplementary Fig. 2E). Interestingly, in contrast to HLC D20, HC3X D20 were able to survive in the absence of glucose (Fig. 2c). In accordance, glucose consumption and lactate secretion were reduced, whereas pyruvate uptake was increased (Fig. 2d). However, no glucose secretion (Fig. 2d) or increased OCR (Fig. 2e) was observed.

As HC3X D20 could be cultured in the absence of glucose, we next evaluated which nutrients fueled the central carbon metabolism of PSCs, HLC D20, and HC3X D20 in vitro. We cultured the different cell populations with ^13^C-labeled tracers for glucose, glutamine, and pyruvate, and measured their contribution to glycolytic (pyruvate, lactate, and alanine) and tricarboxylic acid cycle (TCA) (alpha-ketoglutarate, succinate, and fumarate) metabolites. Undifferentiated HLC (D0) and HLC D20 showed high glucose-derived ^13^C labeling of glycolytic metabolites and high glutamine-derived ^13^C labeling of TCA metabolites (Fig. 2e). Consistent with the consumption data, the contribution of ^13^C pyruvate was significantly increased in all intermediates in HC3X D20, which was even more pronounced upon glucose withdrawal (Fig. 2f). Hence, although overexpression of hepatic TFs could induce a metabolic switch from a glucose to a pyruvate-fueled metabolism, they could not induce a gluconeogenic and oxidative phenotype, and CYP450 function was only modestly increased.

### Amino acid levels are limiting in hepatic culture media

**Fig. 3:**
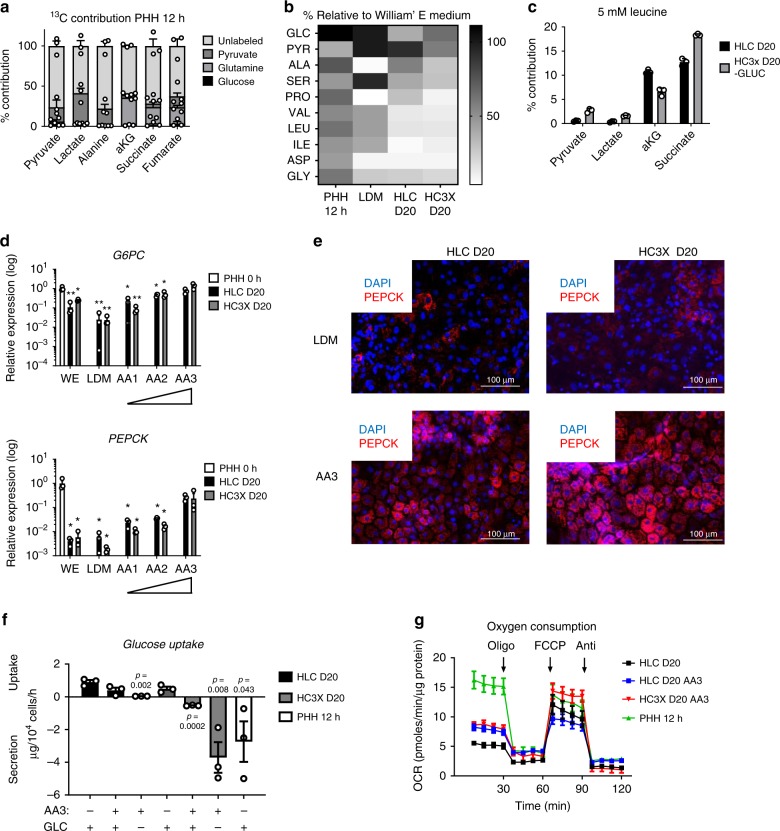
AA supplementation induces metabolic maturation in HLC- and HC3X D20. **a** Percentage of labeling derived from ^13^C-labeled glucose, glutamine, or pyruvate in PHH 12 h. *N* = 3 donors. **b** Relative concentrations of glucose, pyruvate, and the amino acids alanine (ALA), serine (SER), proline (PRO), valine (VAL), leucine (LEU), isoleucine (iLEU), aspartate (ASP), and glycine (GLY) in culture medium of PHH 12 h, HLC D20, or HC3X D20. *N* = 3 independent differentiations. *N* = 3 for each of 2 PHH donors. **c** Percentage of contribution of 5 mM of ^13^C leucine in HLC D20 and HC3X *N* = 3 independent differentiations. **d** Relative gene expression analysis for *G6PC* and *PEPCK* for HLC D20 and HC3X D20 compared with PHH 0 h. Cells were cultured in either WE or LDM supplemented with increasing amounts of amino acids (*N* = 3 independent differentiations. *N* = 4 donors for PHHs). Significance was calculated compared with PHH 0 h grown in William’s E (WE) by unpaired two-tailed Student’s *t*-test. *p < 0.05, **p < 0.01. **e** Representative immunofluorescence images of two independent differentiations for PEPCK staining of HLC D20 and HC3X D20 cultured in LDM or AA3. **f** Glucose uptake or secretion in HLC D20 and HC3X D20 cultured in control medium or AA3 with or without glucose (GLC). Secretion rates for PHH 12 h were used as reference and repeated from Fig. 1f. *N* = 3 independent differentiations, *N* = 3 donors for PHHs. Significance was calculated by comparing HLC D20 in LDM with glucose (GLC) and samples of HLC or HC3X cultured with or without AA3 and with or without GLC by using unpaired two-tailed Student’s *t*-test. **g** Oxygen consumption measurement with addition of oligomycin, FCCP, and antimycin for HLC D20 and HC3X D20 cultured with AA3 without glucose. Data for HLCs and PHH 12 h was used as a reference and repeated from Fig. 1g. N = 6 wells, 5 time point measurements per well. PHH = *N* = 6 wells for 2 different donors, 5 time point measurements per well. Data in all panels represent mean ± SEM with *P*-values indicated when significant. Source data are provided as a Source Data file.

To compare the glucose and pyruvate dependency of HLC D20 and HC3X D20 with the nutrient requirements of PHHs, we next performed the same tracer studies on PHHs. Interestingly, PHHs were found to be fueled mainly by unlabeled carbon sources (Fig. 3a). This led us to the hypothesis that AAs, the only other available carbon source, may represent a major carbon fuel for hepatocytes. Consistent with this, medium recovered following 24 h of PHH culture in William’s E (WE)-based hepatocyte maintenance medium was significantly depleted of pyruvate and several AAs. Surprisingly, this was even more pronounced for HLC D20 and HC3X D20 cultured in the AA-poor liver differentiation medium (LDM) (Fig. 3b). Therefore, we hypothesized that lack of AAs was responsible for the observed glucose or pyruvate dependency. To demonstrate that HLC D20 and HC3X D20 preferentially catabolize AAs, we performed labeling studies with ^13^C leucine (5 mM). In both cell models, we detected extensive^13^C labeling of TCA metabolites and, to a lesser extent, of glycolytic metabolites (Fig. 3c). Based on these results, we cultured HLCs and HC3X progeny in the AA-rich WE-based hepatocyte maintenance medium used commonly to culture PHHs or we supplemented LDM medium with increasing concentrations of AAs (Supplementary Table 1). Surprisingly, only the supplementation of AAs to levels well beyond those present in WE medium increased transcripts for gluconeogenesis genes in both HLC D20 and HC3X D20 (Fig. 3d). Only in AA3 medium, containing five times higher concentrations of AAs than in WE medium, ±100% of cells stained positive for PEPCK (Fig. 3e). When differentiated in AA3 medium, HC3X D20 produced and secreted glucose (Fig. 3f), even if this was not seen for HLC D20. Furthermore, ^13^C-tracing studies suggested a switch towards AA catabolism as observed in PHHs (Supplementary Fig. 3A). In addition, only when HLC D20 and HC3X D20 were cultured in AA3 medium, we observed AA uptake and urea secretion rates that were equal to PHH 12 h (Supplementary Fig. 3C, D).

As AAs induced transcriptional upregulation of gluconeogenic genes, we hypothesized that they not only served as carbon sources but might act as active drivers of the hepatic phenotype. However, when we measured the composition of the interstitial fluid (IF) of the mouse liver, we found the concentration of most AAs to be higher than in serum or LDM, but lower than in AA3 medium, whereas three AAs (glutamine, alanine, and glycine) were present at levels similar to those in AA3 medium (Supplementary Fig. 3B, C). As only the AA3 formulation could double the basal OCR (Fig. 3g), we believe this data suggests that in a static in-vitro differentiation system, AA levels need to be increased above in-vivo physiological levels to induce full metabolic maturation.

### Elevating AA levels induces hepatic maturation

**Fig. 4:**
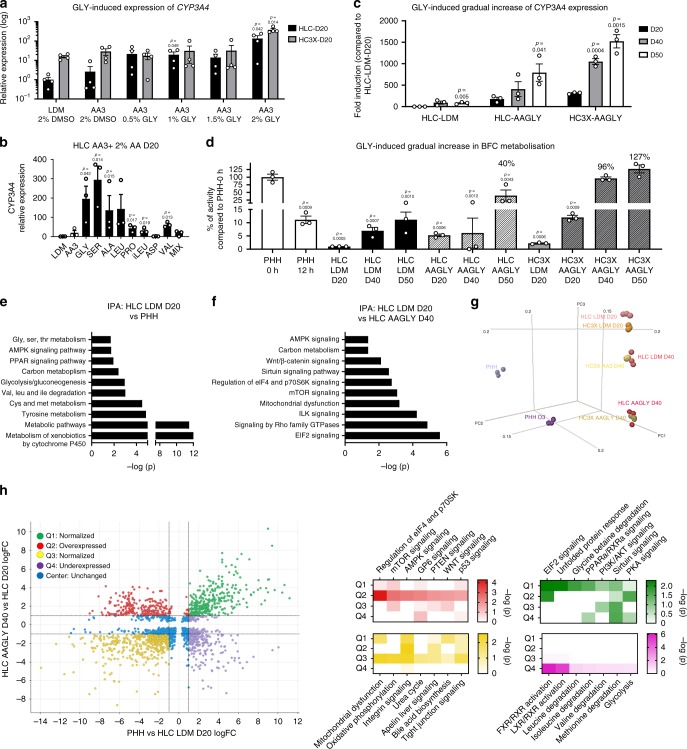
Elevating AA levels induces time-dependent maturation of HLC and HC3X progeny. **a**–**c** Relative *CYP3A4* expression in (**a**) HLC D20 and HC3X D20 cultured in control medium and in medium supplemented with AA3, and an increasing amount of glycine; (**b**) in HLCs and HC3X cultured in control and in AAGLY medium at D20, D40, and D50 of differentiation; (**c**) in HLCs cultured in medium supplemented with AA3 with or without 2% of either glycine (GLY), serine (SER), alanine (ALA), leucine (LEU), proline (PRO), isoleucine (iLEU), aspartate (ASP), valine (VAL), or a mix of all of the above. *N* = 3 independent differentiations. Significance was calculated compared with HLC or HC3X LDM D20 with 2% DMSO by unpaired two-tailed Student’s *t*-test. **d** Relative CYP3A4/CYP2E1-dependent BFC metabolization for HLCs and HC3X grown in different media conditions at different times of differentiation and compared with PHHs. *N* = 3 independent differentiations. *N* = 4 donors for PHH 0 h and PHH 12 h. Significance was compared with PHH 0 h by unpaired two-tailed Student’s *t*-test. **e**, **f** IPA analysis showing major differentially expressed pathways when comparing HLC LDM D20 with (**e**) PHH 0 h and (**f**) HLC AAGLY D40. **g** PCA plot showing the distribution of HLCs and HC3X differentiated in different media and compared with PHH 0 h. **h** Quadrant analysis showing pathways that are normalized (Q1 and Q3), overexpressed (Q2), or underexpressed (Q4) when comparing HLC LDM D20 with PHH 0 h and HLC AAGLY D40. Color intensity correlates with −log(*p*). Data in all panels represent mean ± SEM with *P*-values indicated when significant. Source data are provided as a Source Data file.

Surprisingly, despite the clear correlation between metabolism, AA catabolism, and *CYP450* expression observed in the WGCNA (Fig. 1f, g), AA3 only marginally induced the expression of *CYP3A4* (Fig. 4a). However, AAs were found to drive metabolic maturation in a concentration-dependent manner (Fig. 3d) when exceeding the nutritional need (Supplementary Fig. 3B, C). As glycine and alanine concentrations greatly exceeded those of other AAs in the mouse liver IF, we hypothesized that additional supplementation of, e.g., glycine or alanine might induce further maturation. We observed a concentration-dependent increase in *CYP3A4* expression when HLC D20 or HC3X D20 were differentiated in AA3 medium supplemented with 2% glycine (AAGLY) (Fig. 4a). Furthermore, we achieved a similar induction through the addition of 2% serine, alanine, or leucine, whereas *CYP3A4* was significantly less induced following supplementation with proline, isoleucine, and valine, or a mixture of AAs that together constituted 2% (Fig. 4b). Of note, maturation was only achieved when the 2% dimethylsulfoxide (DMSO) present in the media was replaced by 2% of a single AA, as the combination increased the osmolarity of the media to lethal levels (Supplementary Fig. 4A). As aside from *CYP3A4*, also other *CYP450* isoforms were induced in AAGLY medium (Supplementary Fig. 4B), AAs represent a valuable tool to induce CYP450 enzymes. Importantly, AAGLY medium induced a gradual maturation of HLC and HC3X progeny, as extended culture resulted in a continuous increase in *CYP3A4* expression (Fig. 4c) and function (Fig. 4d) to levels that were in the range of gold standard, freshly thawed PHHs.

**Fig. 5:**
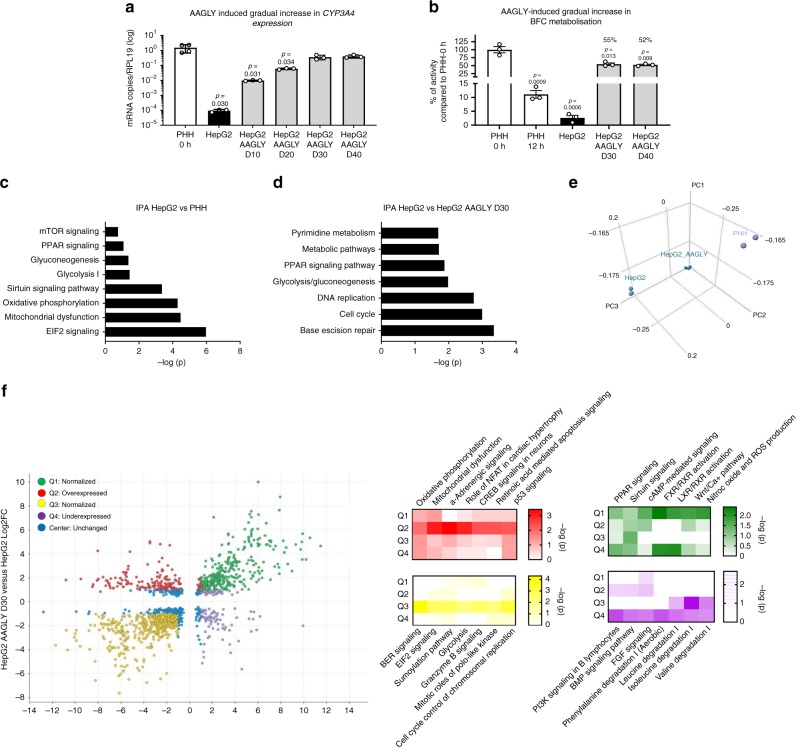
High amino acid concentrations allow for further maturation over time of CYP450 enzymes in HepG2. **a** Gene expression analysis for *CYP3A4* in HepG2 grown for an extended time in AAGLY-supplemented medium and compared with freshly thawed PHHs. *N* = 3 independent maturations of HepG2 cells. *N* = 4 donors for PHHs. Data on PHHs was repeated from Fig. 4. Significance was calculated by comparing with PHH 0 h by unpaired two-tailed Student’s *t*-test. **b** Relative BFC metabolization by HepG2 grown in AAGLY-supplemented medium for 30 or 40 days and compared with PHH 0 h and PHH 12 h. *N* = 3 independent maturations of HepG2 cells. *N* = 4 donors for PHHs. Data on PHHs was repeated from Fig. 4. Significance was calculated by comparing with PHH 0 h by unpaired two-tailed Student’s *t*-test. **c** IPA comparing HepG2 with PHHs. **d** IPA comparing HepG2 with HepG2 AAGLY D30. **e** PCA plot showing three samples of HepG2 grown in either standard medium (HepG2) or AAGLY-supplemented medium (HepG2 AAGLY) and three donors of PHH 0 h. **f** Quadrant analysis showing pathways that are normalized (Q1 and Q3), overexpressed (Q2), or underexpressed (Q4) when comparing HepG2 with PHH 0 h and HepG2 AAGLY D30. Color intensity correlates with −log(*p*). Data in all panels represent mean ± SEM with *P*-values indicated when significant. Source data are provided as a Source Data file.

To determine whether AAGLY medium induced global maturation, we next compared the transcriptome of HLCs and HC3X cells differentiated in the different media with that of PHHs. The top deregulated pathways when comparing HLC LDM D20 with PHHs contained several genes involved in AA catabolism, glucose metabolism, metabolic pathways, and metabolism of xenobiotics by CYP450 enzymes (Fig. 4e). The same analysis, comparing HLC LDM D20 cells with HLC AAGLY D40 cells, showed AAGLY culture medium to induce several metabolic signaling pathways (including mammalian target of rapamycin (mTORC1) and AMP-activated protein kinase (AMPK)) (Fig. 4f). In general, we found both HLC D40 and HC3X D40 cells cultured in AAGLY media to cluster and move increasingly closer to PHHs (Fig. 4g). As AAGLY conditions regulated the expression of a large number of genes (Supplementary Fig. 4C), we next stratified these into four quadrants. Q1 and Q3 represented genes with similar expression in HLC AAGLY D40 cells and PHH, but not in HLC LDM D20 cells (normalized); Q2 represented genes that were induced to levels higher than that found in PHHs (overexpressed); and Q4 represented genes that remained less expressed in HLC AAGLY D40. As shown in Fig. 5h, normalized pathways were linked to metabolic regulation and mitochondrial function. Overexpressed pathways contained metabolic signaling pathways, whereas AA catabolism pathways were still expressed lower than in PHH. These results show the addition of AAs in the culture media is necessary to normalize the hepatic profile through metabolic signaling pathways.

### Amino acids regulate the hepatic transcriptome

To further substantiate the hypothesis that AA levels and metabolism are a direct determinant of hepatic functionality, we tested whether we could use AAs to drive maturation of other hepatic models. We found AAGLY to induce several *CYP450* isoforms in the HepG2 and HUH7.5 hepatic cell lines (Supplementary Fig. 5A), whereas again glycine could be replaced by serine, alanine, or leucine (Supplementary Fig. 5B). Of note, we also found an AAGLY-mediated induction of *CYP1A2* and *CYP2E1* in the embryonic kidney line, HEK293 (Supplementary Fig. 5A). As both isoforms are expressed in kidney cells^32^, this may suggest that AAs might also regulate CYP450 isoforms in other cell types. Although AAGLY medium slowed down the proliferation of several hepatic cell lines, only HepG2 could be maintained long-term in AAGLY medium as a uniform monolayer (Supplementary Fig. 5C). As we observed for HLCs, AAGLY also induced a gradual increase in CYP3A4 expression (Fig. 5a) and function (Fig. 5b) of HepG2 cells over time.

Kyoto Encyclopedia of Genes and Genomes (KEGG) analysis of the differentially expressed genes (DEGs) in the transcriptome of HepG2 vs. PHHs revealed that the immature phenotype observed in HepG2 was also linked to metabolic pathways, mitogen-activated protein kinase (MAPK) and peroxisome proliferator-activated receptor (PPAR) signaling, and AA catabolism (Fig. 5c). As in the HLC model, AAGLY supplementation resulted in a global improvement of the HepG2 transcriptome (Fig. 5d), specifically by modifying the expression of metabolic and proliferative pathways (Fig. 5e). The quadrant analysis showed metabolic and WNT signaling, combined with pathways involved in cell cycle control and glycolysis to be normalized following culture of HepG2 in AAGLY (Q1 and Q3). Overexpressed pathways contained mitochondrial signaling and OXPHOS genes (Q2), whereas pathways that were still less expressed in HepG2 AAGLY D30 vs. PHH D0 mainly consisted of AA catabolism genes (Q4). Thus, culture of HepG2 in AAGLY medium phenocopied the majority of metabolic maturation features also observed in HLC and HC3X (Fig. 5f).

### Amino acids drive differentiation through metabolic signaling

**Fig. 6:**
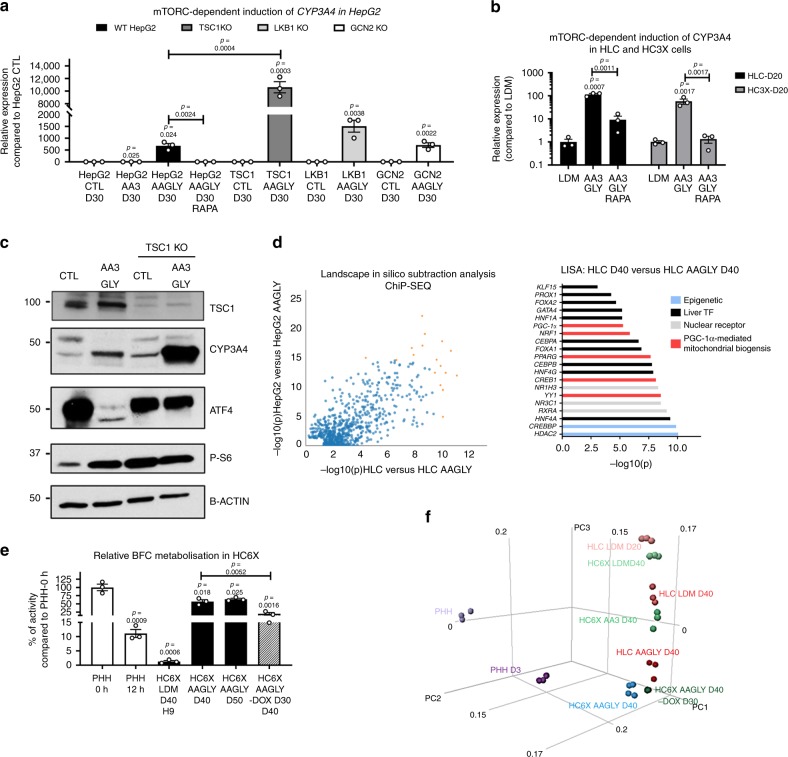
AAGLY-mediated maturation is dependent on activation of mTORC1. **a** Relative expression of *CYP3A4* in HepG2 differentiated with AAGLY, in combination with rapamycin addition (RAPA), or using HepG2 wherein *TSC1*, *GCN2*, or *LKB1* were knocked out. *N* = 3 independent differentiations. Significance was calculated by comparing against HepG2 CTL. Significance was also calculated between Hepg2 AAGLY D30 and HepG2 AAGLY RAPA, and between HepG2 AAGLY D30 and TSC1 KO AAGLY D30 when indicated. Significance was calculated by unpaired two-tailed Student’s *t*-test. **b** Relative gene expression analysis for *CYP3A4* in HLC and HC3X differentiated with AAGLY until D20, with or without rapamycin (RAPA). Expression of HLCs and HC3X cells was related to differentiations in LDM. *N* = 3 independent differentiations. Significance was calculated by comparing against the LDM and between LDM AA3GLY and LDM AA3GLY RAPA by two-tailed unpaired Student’s *t*-test. **c** Western blotting analysis comparing the phosphorylation of S6 and the expression of ATF4 and CYP3A4 in HepG2 differentiated in control media or AAGLY media, with or without KO of *TSC1*. Representative blot of two repeats. **d** LISA analysis identifying the top TFs responsible for the genome wide difference between HepG2 and HLC grown in control or AAGLY media. **e** BFC metabolization comparing HC6X LDM D40 and HC6X AAGLY D40/50, as well as HC6X AAGLY D40 where doxycycline was removed from d30 onwards (HC6X AAGLY D40-DOX from D30). *N* = 3 independent differentiations. N = *3* donors for PHHs. Significance was calculated as compared with PHH 0 h by unpaired two-tailed Student’s *t*-test. Significance was also calculated between HC6X AAGLY D40 and HC6X AAGLY –DOX D30-D40. **f** PCA plot showing the distribution of HLCs, HC3X, and HC6X differentiated in different media and compared with PHHs. Data in all panels represent mean ± SEM with *P*-values indicated when significant. Source data are provided as a Source Data file.

Based on the RNAseq data, we next analyzed the activity of the AKT-mTORC1, AMPK, and eIF2alpha pathways in HepG2 cultured with AA3 ALA or AA3GLY (strong inducers of *CYP3A4*) vs. AA3 VAL (weak induction of *CYP3A4*). This analysis linked induction of mature hepatic markers such as CYP3A4, PEPCK, and glutamine synthetase to an increased phosphorylation of S6, AMPK, and AKT. Although we did not find differences in eIF2alpha phosphorylation, we observed a decrease of the eIF2alpha target gene and AA deprivation factor ATF4 (ref. ^33^) (Supplementary Fig. 6A). From these pathways, we were able to specifically link mTORC1 signaling to the induction of CYP3A4, as addition of the mTORC1 inhibitor rapamycin blocked the induction of CYP3A4, while AMPK phosphorylation and ATF4 levels remained unchanged (Supplementary Fig. 6A). Furthermore, knockout (KO) of the upstream regulators of AMPK and eIF2alpha, *LKB1* and *GCN2*, did not affect CYP3A4 levels both in control media or AAGLY media (Supplementary Fig. 6B, C). In both HepG2 (Fig. 6a), HLC and HC3X (Fig. 6b), mTORC1 appeared to regulate *CYP3A4* expression rather than translation. Finally, although KO of the mTORC1 inhibitor Tuberous sclerosis (*TSC)1* was not sufficient for driving maturation in HepG2, culture of *TSC1* KO HepG2 in AAGLY medium yielded an even greater induction of *CYP3A4* compared with wild-type HepG2 (Fig. 6a, c). This data suggests a crucial, but not sufficient role, for the activation of mTORC1 in inducing CYP3A4.

To identify downstream targets of mTORC1, we performed a landscape in-silico subtraction analysis (LISA)^34^. This demonstrated that AAGLY-mediated maturation is likely driven by hepatic TFs and genes involved in PGC-1α-dependent mitochondrial biogenesis (Fig. 6d). PGC-1α-mediated mitochondrial biogenesis was recently described as an essential regulator of hepatic maturation^28^, represents a cofactor for PPARs^35^, and is regulated by mTORC1 (ref. ^36^), sirtuins (SIRT)^37^ and AMPK^38^. As our transcriptome analysis showed normalization of mitochondrial function (Figs. 4g and 5g), we subsequently tested if AAGLY-induced differentiation was correlated with mitochondrial biogenesis. We found that supplementation with AAGLY induced a significant increase in HepG2 cell size and mitochondrial mass (measured by the corrected total cell fluorescence (CTCF) for the mitochondrial import receptor subunit TOMM20 homolog (TOMM20)) (Supplementary Fig. 6C). Moreover, the activity of the electron transport chain (ETC) and the mitochondrial enzyme, citrate synthase, were all induced by AAGLY supplementation (Supplementary Fig. 6D). To determine whether the effect on mitochondrial biogenesis and hepatic maturation induced by AAGLY could be replaced by incorporation of TFs induced by AAGLY supplementation, we integrated an inducible *PGC-1α* (PGC1A cells) cassette alone or a cassette containing the three inducible TFs already present in the HC3X line, together with an inducible cassette containing the *PGC-1α* complementary DNA, as well as the cDNA for its posttranscriptional regulator *SIRT1* and a constitutively active variant of *AMPK* (HC6X). Addition of doxycycline induced transcript levels of *PGC-1α* (PGC1A cells) alone or all six TFs (HC6X) near levels seen in PHHs. However, expression of the ETC components such as cyclooxygenase *(COX)1*, cytochrome *(CYT)C*, and ATP synthase *(ATP)5A1* were only induced in HC6X progeny and not PGC-1α progeny (Supplementary Fig. 6F). In HC6X D40, cultured in LDM, we observed an induction in mitochondrial mass similar to that of HLCs or HC3X cultured in AAGLY medium (Supplementary Fig. 6G). However, we only found an increase in *CYP3A4* and the gluconeogenic genes, *G6PC* and *PEPCK*, when HC6X were also cultured with AAGLY, indicating that mitochondrial biogenesis is not sufficient to drive maturation (Supplementary Fig. 6E, F). The transcriptome of HC6X AAGLY D40 was similar to that of HC3X AAGLY D40, even if perhaps slightly closer to that of PHHs (Fig. 6f). Of note, removal of doxycycline from D30 onwards (HC6X AAGLY D40-DOX from D30) caused a significant decrease in CYP3A4 activity (Fig. 6e) and to a lesser extent also decreased global maturity (Fig. 6f). These results indicate that although overexpression of hepatic TFs only has a minimal effect in the context of standard culture media, AAGLY conditions allow hepatic TFs to drive maturation. Indeed, a LISA analysis comparing HC6X AAGLY D40 with HC6X AAGLY D40-DOX from D30 showed that the major difference between both cell populations is due to the activity of hepatic TFs (Supplementary Fig. 6G).

### AAGLY-mediated maturation enables identification of hepatotoxicants

**Fig. 7:**
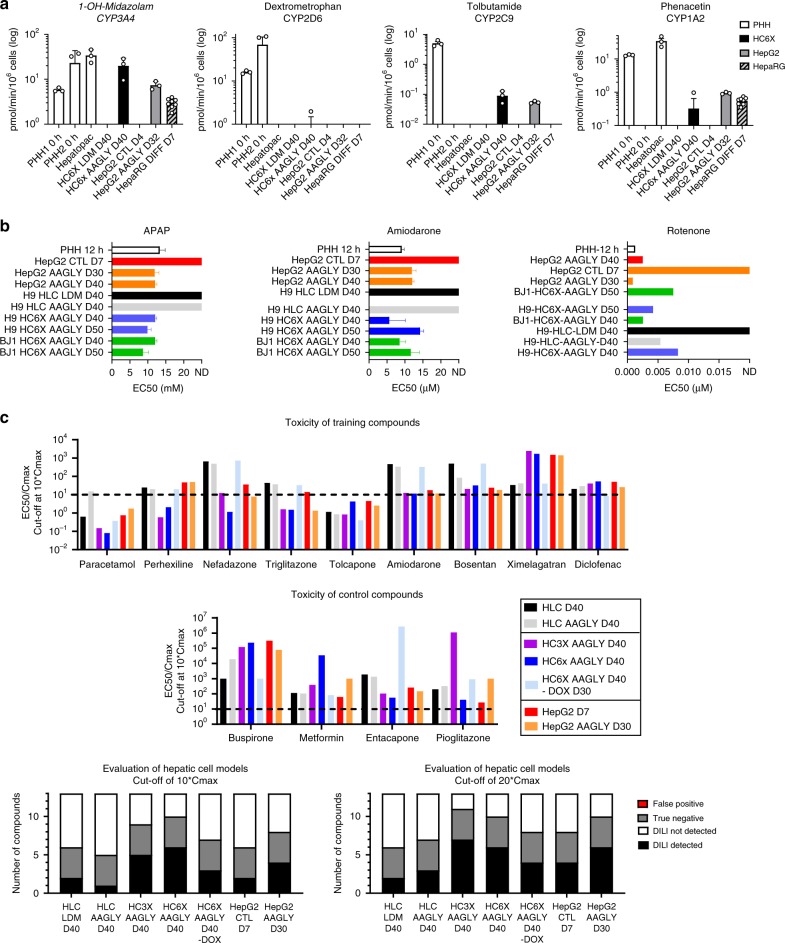
AA supplementation allows for hepatotoxic drug screening in a high-throughput format. **a** CYP3A4-dependent formation of 1-OH midazolam and CYP450-dependent biotransformation of dextromethorphan (2D6), phenacetin (1A2), and tolbutamide (2C9) after 1 h of treatment. Treated cells consist of PHHs maintained in suspension from two donors (PHH1 and PHH2) (*N* = 3 thawings), the Hepatopac® system (*N* = 3 donors), HC6X differentiated in either LDM or AAGLY for 40 days (*N* = 3 independent differentiations), and HepG2 in control media (HepG2 CTL D4) or differentiated in AAGLY for 30 days (HepG2 AAGLY D30) (*N* = 3 independent maturations), and HepaRG cells (*N* = 8 differentiations). Undetectable values are not shown on the logarithmic scale. **b** EC50 values for amiodarone, APAP, and rotenone for cells cultured in control or AAGLY medium. Cultures consist of PHHs cultured for 12 h (PHH 12 h), HepG2 cultured for 7 days in control media (Hepg2 CTL D7), or differentiated for 30 days in AAGLY (HepG2 AAGLY D30), HLCs, and HC6X in the H9-ESC or BJ1-iPSC background differentiated for 40 days in either LDM or in AAGLY; *N* = 6 separate well per concentration for treated cells. *N* = 18 separate wells for control cells. One donor of PHHs was analyzed in six wells. Viability was determined by Hoechst and Draq7 staining. **c** EC_50_/*C*_max_ values for nine hepatotoxic and four non-hepatotoxic training compounds. Cell viability was measured by resazurin conversion. Data in all panels represent mean ± SEM. Source data are provided as a Source Data file.

Given the acquisition of a more mature phenotype in both HepG2 cells and PSC- hepatic progeny, we next evaluated the ability of these cells to biotransform drugs and predict drug-induced liver injury (DILI) in comparison with standard models including PHHs^39^, HepaRG cells^9^, and the micropatterned PHH-based Hepatopac® system^5^. First, we evaluated biotransformation of midazolam, dextromethorphan, tolbutamide, and phenacetin by HepG2 AAGLY D32 and HC6X AAGLY D40 after a single exposure. As shown in Fig. 7a, the biotransformation rates for all drugs were found to be in the range of the PHH-based Hepatopac® system and surpassed that of the HepaRG model. More specifically, midazolam biotransformation was equal to that of PHHs, whereas the biotransformation by CYP2D6, CYP1A2, and CYP2C9 were found to be in the range of 1–10% of the best PHH donor^22,40,41^. Importantly, drug biotransformation remained stable for at least 10 days (Supplementary Fig. 7A). In accordance, we found that HC6X AAGLY D40 stained positive for CYP3A4, with several cells displaying a binucleated phenotype (Supplementary Fig. 7B).

To evaluate the ability to predict hepatotoxicity, we adapted the differentiation assays to a 384-well format. First, we tested sensitivity to a single administration of amiodarone and acetaminophen (APAP) (both requiring CYP450-dependent metabolization), and to the mitochondrial toxin rotenone, known to be highly toxic for PHHs. Although no toxicity was seen for HepG2, HLC, and HC6X cultured in basal medium, exposure of all three cell types cultured in AAGLY-supplemented medium to these drugs resulted in sigmoidal kill curves and EC_50_ values that were similar to PHHs, and in line with previously published PHH-based toxicity studies^5,42^. Most importantly, all models were stable, as similar drug toxicity was seen after an additional 10 days of culture. Moreover, generation and differentiation of an HC6X cell line in an iPSC background (BJ1) yielded very similar results (Fig. 7b, Supplementary Fig. 7C, and Supplementary Table 2). We also addressed the ability of HepG2, HLC, HC3X, and HC6X, cultured in basal medium or medium supplemented with AAGLY, to predict DILI. We used the protocol described by Sison-Young et al.^43^, based on repeated administration of 13 gold standard DILI training compounds. As a readout, we evaluated cell viability by resazurin conversion and correlated the results to therapeutic doses (*C*_max_ values). As shown in Fig. 7c and Supplementary Fig. 7C, we found HC6X AAGLY D40 or HC3X AAGLY D40 to perform the best when using an EC_50_/*C*_max_ cut-off = 10 or 20, respectively. Although AAGLY also increased sensitivity in the HepG2 model, here several drugs were still identified as false negative. Last, removal of doxycycline in the last 10 days of differentiation had a detrimental effect on drug sensitivity (HC6X AAGLY D40 vs. HC6X AAGLY D40-DOX from D30). This reinforces the notion that a combinatorial approach of metabolic and genome engineering creates the most optimal PSC progeny (Fig. 7c and Supplementary Fig. 7C). In conclusion, AAGLY-mediated maturation greatly increases drug sensitivity in both HepG2 and PSC-based models, but only the HC3X AAGLY D40 and HC6X AAGLY D40 cells were found to identify hepatotoxicants to a similar extent as PHHs^43^.

## Discussion

In this study, we identified AAs as important regulators of hepatic metabolism and function in an in-vitro setting. Specifically, we demonstrate that elevating AA levels beyond the nutritional need can transcriptionally drive the transformation from a glucose-dependent to an AA-fueled and glucose-producing metabolic profile. Furthermore, we show that AAs feed into the regulation of the hepatic transcriptional network and thus drive, in a manner that is similar to growth factors, hepatic differentiation. As a consequence, AAs can be used as a tool to induce maturation of HLCs and hepatic cell lines. Indeed, cells grown in AAGLY conditions acquire the capability to biotransform drugs at levels similar to PHHs^40,41^ and are able to stratify DILI training compounds at similar levels as PHHs^43^. Therefore, we propose nutrient engineering as a complementary strategy to growth factor treatment, ECM engineering, transcription factor overexpression, and organoid cultures in the context of PSC hepatocyte differentiation and maturation^26^.

The observation that nutrients can actively influence cellular maturation is in line with recent publications demonstrating that the removal of glutamine can boost the pluripotency network^44^, that addition of oxidized metabolites can drive differentiation towards the neuronal lineage^27^, and that lactate allows purifying cardiomyocytes^45^. In the context of hepatocyte differentiation, no such studies have been performed. Hepatocytes are among the metabolically most active cells, containing a highly elaborate mitochondrial network^28^, and mitochondrial dysfunction has been implicated in several liver-related diseases^46,47^. In addition, a few studies have suggested that the overall metabolite concentrations are much higher in adult than fetal cultured hepatocytes^48^, and that cultured PHHs are in an extremely starved state^49^. Our data suggest that HLCs and HepG2 grown in standard conditions indeed experience suboptimal AA levels. However, we also show that full functional maturation and the induction of drug biotransformation requires the addition of AAs beyond the nutritional need of HLCs or HepG2. Thus, although previous studies identified AAs as important for hepatocyte survival, proliferation, and protein synthesis^50,51^, we here demonstrate that increasing AAs to levels 30-fold above that of typical hepatocyte media, actively induces a metabolic switch, as well as CYP450 enzyme expression and function (Supplementary Table 3). Of note, AA levels in the liver IF have been described to exceed that of serum^52^, which we confirmed here, and are believed to fuel glucose, urea, and protein production in a concentration-dependent manner in vivo^53^. Although we demonstrate AA requirements for inducing in-vitro differentiation to be supra-physiological, it is difficult to compare a perfused (in vivo) to a static (in vitro) state. This would be in line with the fact that in many instances supra-physiological concentrations of growth factors are used in vitro^54^, further reinforcing the notion of AAs as signaling molecules. This study therefore demonstrates that AAs represent a tool to manipulate stem cell differentiation, as has already been shown in the cancer metabolism field^55–57^.

Of the investigated signaling pathways, we only found inhibition of mTORC1 to be able to prevent the induction of *CYP3A4* under conditions of AA supplementation. Activation of mTORC1 has been shown to be important for liver regeneration and hepatocyte maturation in vivo^58,59^. Nevertheless, the sole activation of mTORC1 through the genetic deletion of TSC1 was not sufficient to induce *CYP3A4* expression. This finding could be in line with studies demonstrating that *TSC2* KO in mouse embryonic feeders (MEFs) does not cause mTOR/S6K activation when cultured under AA-deprived conditions^60^, and that mTORC1 signaling requires metabolic fluxes such as autophagic lipid catabolism to sustain mTORC1 signaling, even following genetic ativation^61^. Alternatively, AAGLY likely induces several other metabolic signaling pathways other than mTORC1. In fact, our RNAseq studies demonstrate that AAs have a global effect on hepatic maturation and target multiple aspects of hepatic biology, including WNT, BMP, and cAMP signaling. This result is in accordance with a recent paper identifying a culture system for the long-term maintenance of PHH functionality through the simultaneous pharmacological manipulation of 5 key signaling pathways (TGF-β, Notch, WNT, BMP, and cAMP)^7^. Thus, it appears that at least three of these pathways are not only involved in maintaining a mature hepatocyte phenotype, but also drive maturation of immature cell populations. Moreover, our data suggest that this can be achieved via a nutritional rather than pharmacological approach. To link the activation of these pathways to hepatic maturation, we mapped the TFs that are responsible for the difference between cells differentiated in standard media and in AAGLY. In this analysis, we found liver TFs, such as the CYP450-linked Kruppel-like factor 15 (ref. ^62^) and HNF4α^63^, to be the major regulators. Therefore, we believe that AAGLY conditions are a requirement for the hepatic transcriptional network to drive maturation, whereas the overexpression of TFs has a complementary effect. Last, AAGLY supplementation allows for a gradual maturation of both PSC progeny and HepG2 over 30–40 days. This suggests that the AAGLY supplementation induces several transcriptional and perhaps epigenetic mechanisms that ultimately lead to a phenotypic maturation. Therefore, the low levels of AA in standard media for HLCs and HepG2 cells may represent a block in hepatic maturation.

Finally, we demonstrate that the AAGLY-supplemented models display drug biotransformation that approaches that of the current gold standard PHHs^39,64^. We also observed that especially the combined genome and nutrient engineering strategy allowed for the best prediction of DILI. HC3X- and HC6X AAGLY D40 cell correctly identify seven out of nine hepatotoxins (at EC_50_/*C*_max_ = 20). These results are similar to the average of the seven PHH populations described by Sison-Young et al.^43^. In contrast to PHHs cultured in classical 2D culture conditions, we also show that both HepG2 and HC6X differentiated in AAGLY medium maintain stable drug biotransformation and drug sensitivity for at least 10 days, indicating their suitability for repeat dose toxicity and long-term drug biotransformation studies. In conclusion, this study highlights nutrients as a potential tool to guide hepatic maturation in several cell models. The optimization described creates significantly improved and long-term stable models for studying hepatocyte biology, drug biotransformation, and identification of liver toxicants.

## Methods

### hPSC differentiation to the hepatocyte lineage

The human embryonic stem cell (hESC) line H9 (WA09) was purchased from WiCell Research Institute (Madison, 15 WI) and kept according to supplier’s instructions as feeder-free cultures on Matrigel (BD biosciences)-coated plates in Essential 8 (or Essential 8 Flex) (Thermo fisher). BJ1 iPSCs were made in-house and kept on Matrigel-coated plates at ±8.75 × 10^4^ cells/cm^2^ in mTeSR medium (Stem Cell Technologies)^65^. Cells were differentiated towards the hepatocyte lineage as described by our group before^65^, with some optimizations. Briefly, single-cell suspensions of H9/ BJ1 cells (using accutase) were plated on Matrigel-coated plates at ±8.75 × 10^4^ cells/cm^2^ in mTeSR medium (Stem Cell Technologies). When cells reached 70–80% confluency, differentiation was started using the previously described cytokine regimes and was stopped after 20 days of differentiation. All cytokines were purchased from Peprotech (NJ). Differentiation medium was supplemented with 0.6% DMSO during the first 12 days of the culture and with 2% DMSO during the last 8 days of differentiation. Use of embryonic stem cells and iPSCs for research was approved by the “Commissie Medische Ethiek,” UZ KU Leuven/Onderzoek U.Z. Gasthuisberg, Herestraat 49, B 3000 Leuven, under file number S52426

### Primary cells

As positive control for hepatic expression and function, two donors (C304 (male, 64 years of age) and C303 (female, 52 years of age)) of crypopreserved and platable PHHs were bought from Corning Biosciences (Corning® Gentest™ Plateable Human CryoHepatocytes, Metabolism-Qualified, ≥5 million cells (Product #454543)). Furthermore, two additional donors (donor F125 (male, 62 years of age) and donor F110 (female, 26 years of age)) were obtained from the Ministery of Health-accredited tissue banks at the Cliniques Univeristaires St Luc, Brussels. This post-mortem collected tissue was obtained under the Belgian legislation on organ and tissue donation. The organ collected within the opting out system supposes that the deceased donor of his representative has made no opposition for organ donation, including for research purpose. All four donors were used for gene expression assessment, whereas Seahorse and toxicity experiments were performed on donor C303 and F125. Functional characterization was performed on donors C3030, F125, and C303. Primary cells were thawed using the Corning® Gentest™ High Viability CryoHepatocyte Recovery Kit (Corning) and plated on collagen I-coated plates.

### Recombinase-mediated cassette exchange

The master cell lines suitable for RMCE were generated using zinc-finger mediated integration of a flippase recombinase target-flanked donor cassette into the *AAVS1* locus and are available^31^. RMCE was performed by nucleofection of the master cell line with a donor vector and the FLPe-expressing vector. Nucleofection was done on 3 million cells obtained after accutase treatment using the hESC Nucleofector Solution Kit 2 (Amaxa) and program F16 using an Amaxa nucleoporator. Cells were plated on puromycin-resistant MEFs (Stem Cell Technologies, Puromycin-Resistant Mouse Embryonic Fibroblasts, Day E13.5) in hESC medium^66^. Donor plasmids were generated through Gibson assembly (NEB) of PCR-amplified open reading frames of the desired genes. Plasmids were then evaluated by digestion and Sanger sequencing. The constitutively active AMPK construct was bought from Addgene (plasmid number 60127) and was generated by the lab of Professor Morris (University of Pennsylvania). The flow cytometry gating strategy for evaluating loss of green fluorescent protein signal is illustrated in Supplementary Fig. 8

### Media optimization and composition

Differentiation of PSCs (and genetically modified PSCs) was performed in LDM as previously described^65^ (the composition is listed in Supplementary Table 4). ^13^C-labeled carbon sources were added to medium, made from powder of Dulbecco’s modified Eagle’s medium (DMEM) without glucose, l-glutamine, phenol red, sodium pyruvate, and sodium bicarbonate. As Molecular, Cellular, and Development Biology (MCDB) media also contains glucose, the MCDB component of LDM was replaced by DMEM.

AA supplementation was accomplished through the addition of MEM Non-Essential Amino Acids Solution (100 × ) and MEM Amino Acids Solution (50 × ) (Thermo Scientific) to the culture medium. For AA1, AA2, and AA3 medium, we added respectively 2 ml, 8 ml, and 16 ml of non-essential AA solution and 1 ml, 4 ml, and 8 ml of essential AA solution per 100 ml of LDM, with or without glucose. Culture medium was then made PH neutral through the addition of NaOH. Addition of glycine or any other of the single AAs was accomplished by adding 20 g/l of each AA powder (Sigma). PSC derived HLCs were differentiated in LDM until D12. AA supplementation (AA1, AA2 and AA3) was performed from day 12 until day 20, 30 or 40 of differentiation. Removal of glucose and/or addition of glycine (or other single AAs) at 20 g/l was done from D14 onwards. Upon addition of glycine, DMSO was removed from the culture media. HepG2, HUH7.5, Hep3B, HHL5, and HEK293 cells were maintained in DMEM supplemented with 10% fetal bovine serum as shown in Supplementary Table 4. AA supplementation consisted of addition of 16 ml of non-essential AA mix and 8 ml of essential AA mix per 100 ml of medium and a further addition of 20 g/l of any of the single AAs. Hepatoma cell lines were plated in normal culture medium, with addition of AA3 + 20 g/l AA once cells reached 60% confluency, and this for 3 or 7 additional days of culture. PHHs were maintained in a medium composition that was based on WE as detailed in ref. ^67^.

### Intracellular AAT flow cytometry

Single-cell suspensions of d20 PSC progeny (following trypsinization with 0.25% Trypsin) were fixed with 4% paraformaldehyde (PFA) for 15 min, permeabilized with 0.1% saponin for 15 min, and blocked with 10% goat serum (Dako, 15 Glostrup, Denmark) for 45 min, all at room temperature. Samples were stained using 0.0625 µg/200 µl of the anti-AAT antibody (Dako) for 1 h followed by an Alexa Fluor 647 secondary antibody (1:1500) (Invitrogen) for 30 min and analyzed using a FACS-Canto (BD Biosciences). As isotype control, we used rabbit IgG (BD Pharmingen). The gating strategy is illustrated in Supplementary Fig. 9.

### Immunofluorescence staining

Cells were fixed with 4% PFA for 15 min at room temperature. After permeabilization with 0.2% Triton X-100 for 15 min, samples were blocked with 10% donkey goat serum (Dako, 15 Glostrup, Denmark) for 15 min. Staining with the primary antibodies and isotype controls was performed overnight at 4 °C. Next, the samples were stained using the corresponding secondary antibodies for 1 h and with Hoechst (Sigma-Aldrich) for 10 min. An overview of all used antibodies and their working concentration is given in Supplementary Table 5. Images were taken using an Axioimager.Z1 microscope (Carl Zeiss). Intensity of TOMM20 staining was measured using the ImageJ Mito-Morphology Macro^68^ and is shown as the CTCF, with CTCF = Integrated Density − (Area of selected cell × Mean fluorescence of background readings).

### RNA extraction and quantitative reverse-transcription PCR

RNA extraction was performed using the GenElute Mammalian Total RNA Miniprep Kit (Sigma-Aldrich). Then, genomic DNA was eliminated using the On-Column DNase I Digestion kit (Sigma-Aldrich). Last, 1 µg of RNA was transcribed to cDNA using the Superscript III First-Strand synthesis (Invitrogen). Gene expression analysis was performed using the Platinum SYBR green qPCR supermix-UDG kit (Invitrogen) in a ViiA 7 Real-Time PCR instrument (Thermo Scientific, Waltham, MA). The sequences of all used qRT-PCR primers are listed in Supplementary Table 6. Of these, ribosomal protein L19 (*RPL19*) was used as a housekeeping gene for normalization.

### RNAseq analysis

RNAseq analysis was performed by the VIB Nucleomics core (Leuven, Belgium). RNAseq fastq files were collected, and low-quality ends and adapter sequences were trimmed off from the Illumina reads using trimmomatic. Processed reads were mapped and aligned with Spliced Transcripts Alignment to a Reference (STAR) to the Human reference genome (GRCh38.91) with default ENCODE parameter settings, except for “--runThreadN 16 --sjdbOverhang 76.” Reads per kilobase of transcript per million mapped reads were computed using Cufflinks and then normalized to transcripts per million (TPM). Using R Studio, TPM was then log2 transformed before performing hierarchical clustering using Euclidean distance and centroid linkage by unweighted centroid clustering. Principal component analysis (PCA) was done using the “ggplot2” package in statistical software, R. HTSeq package with Python was utilized to identify the raw counts inputted into the “EdgeR” package in R to compute differential gene expression. The threshold criteria of *P*-value and false discovery rate (FDR) ≤ 0.05, and a fold change of ≥2 in gene expression among the different pairwise comparisons were used to identify the DEGs. Functional analysis or Gene ontology (GO) of the different DEGs was done utilizing DAVID v6.8, whereas pathway comparison analysis was done in Ingenuity Pathway Analysis. Further visualizations of the data including the PCA plot and the comparative analysis of pairwise comparisons to perform the quadrant analysis were done using TIBCO Spotfire v7.6.1 OmicsOffice package.

### Quadrant analysis

Differential gene expression analysis was done in the statistical software, R (v 3.3.3). Python software htseq-counts was utilized to extract the necessary raw read counts to upload into the “EdgeR” package to determine differential gene expression. A threshold criteria of *P*-value and FDR ≤ 0.05, and a fold change of ≥2 in gene expression among the different pairwise comparisons were then used to identify the significant DEGs. Visualizations of the pairwise comparisons of DEGs were elucidated in a quadrant comparative analysis done in TIBCO Spotfire v7.6.1. Data were first filtered so that the *P*-value and FDR were ≤0.05 for each pairwise comparison before being represented in a scatter plot.

### Weighted gene correlation network analysis

We queried ArrayExpress for microarrays of both stem cell-derived hepatocytes and primary hepatocytes. Only samples that were not of cancerous origin, and that were labeled with biotin, were used for analysis. Samples were robust multichip average normalized per platform, followed by quantile normalization to remove platform effects. Probe intensities were summarized by the geometric mean. Only genes that were significantly differentially expressed (Holm–Bonferroni adjusted *p*-value < 0.05) between HLCs and primary hepatocytes were kept for analysis. Furthermore, we applied a cut-off on the expression range. Transcription factors differing less than twofold over all samples were removed, as well as other genes differing less than fourfold over all samples. Using this cleaned dataset, we perform a signed WGCNA analysis as described by Langfelder et al.^29^. A soft-thresholding power of 19 was used as to approach scale-free topology (*R*^2^ = 0.864) and highly correlated modules were merged (cut height 0.2). To identify which pathways were dysregulated in each cluster, we performed GO analysis using the TopGO R package and KEGG pathway-enrichment analysis using the clusterProfiler R package.

### Epigenetic landscape in-silico subtraction analysis

For the LISA analysis, we uploaded the 500 genes with highest differential expression and a *p*-value of at least 0.05 into the LISA cistrome website. Top transcription factors were shown based on −log (*P*) value.

### Seahorse measurements

Oxygen consumption and extracellular acidification were analyzed by differentiating the cells in Seahorse XF24 tissue culture plates (Seahorse Bioscience Europe, Copenhagen, Denmark). The measurement of OCR was performed at 10 min intervals (2 min mixing, 2 min recovery, and 6 min measuring) for 3 h using the Seahorse XF24 analyzer. Cells were exposed to 1 µM of oligomycin (Oligo) to correlate oxygen consumption with mitochondrial activity, to 1.5 µM of FCCP, an uncoupler of the ETC, to measure maximal respiration, and finally, to 1 µM of antimycin (Anti). Data were then normalized for protein content, quantified using the Pierce™ BCA Protein Assay Kit (Thermo Scientific, 23225) following manufacturer’s instructions.

### GC and HPLC MS measurements

Metabolite extraction, derivatization, and measurement on the gas chromatography–mass spectrometry (GC-MS) was done as described previously^69^. Briefly, cellular metabolism was quenched in liquid nitrogen. Metabolites were then obtained through a cold two-phase methanol–water–chloroform extraction and phase separation at 4 °C, dried using a vacuum concentrato, and stored at −80 °C. Sample derivation was accomplished by a reaction with 7.5 µl of methoxyamine (20 mg/ml in pyridine) for 90 min at 37 °C and with 15 µl of *N*-(tert-butyldimethylsilyl)-*N*-methyl-trifluoroacetamide for 60 min at 60 °C. Metabolite levels and isotopomer distribution was then measured with a 7890 A GC system (Agilent Technologies). Isotopomer distributions were extracted from the raw ion chromatograms using a custom Matlab M-file. Isotopomers were then calculated as a fraction of the total amount. Glucose, pyruvate, and lactate concentrations were measured in the medium samples by high-performance liquid chromatography (HPLC). Medium samples were taken at different time points and stored at −80 °C. They were measured on a HPLC Bio Rad organic acid analysis column, Aminex HPX-87H, Ion exclusion column, 300 mm × 7.8 mm with 5 mM H_2_SO_4_ as the mobile phase. Peeks were integrated manually, as was a standard curve for glucose, lactate, and pyruvate made in basal medium. For tissue samples, tissues were weighed (5–10 mg) and pulverized (Cryomill, Retsch) under liquid nitrogen conditions. Next, −20 °C cold 65% methanol was added to the samples, followed by −20 °C cold chloroform. Samples were then processed as described above (GC-MS measurement). For tissue extracts, the total ion counts were normalized to the internal standard and tissue weight.

### Liver IF extraction

The liver IF extraction method has been modified and adapted from Nagahashi et al.^70^ and from Wiig et al.^71^. Briefly, five 9-week-old, healthy Balb/c female mice were killed with 50 µL of 60 mg/ml of Dolethal (pentobarbital sodium). Subsequently, livers were collected by surgical resection, washed in a blood bank saline solution, and dried from liquid excess by tapping in a gauze. Subsequently, the livers were placed in a filtered centrifuge tube created by using a column-centrifuge tube in which the pre-existent filter has been substituted with a nylon mesh filter with 20 µm opening pores (Spectrum Labs, Product code: S145811). The IFs (around 2–5 µL for each organ) was collected in the column-centrifuge tubes by centrifugation at 400 × *g*, 4 °C for 10 min, and processed immediately after extraction. The animal study complies with ethical regulations and was approved by the KU Leuven ethics committee. Housing and experimental animal procedures were approved by the Institutional Animal Care and Research Advisory Committee of KU Leuven, Belgium.

### Generation of HepG2 KO cell lines

KO cell lines were generated by transducing HepG2 cells with lentiviral particles containing the lentiCrispr v2 construct (Addgene Plasmid #52961). After puromycin selection, gene KO was evaluated through western blot. sgRNAs used were 5′-GCACCTTGGTGGATTATTACC-3′ for the *TSC1* KO, 5′-GGAAATGCTGGACAGCGTGC-3′ for *GCN2* KO, and 5′-GGAAATGCTGGACAGCGTGC-3′ for *LKB1* KO.

### Functional assessments

Urea concentrations in the medium were analyzed after 24 h of culture using the QuantiChromTM Urea Assay Kit (BioAssay Systems DIUR-500) according to the manufacturer’s instructions. CYP3A4-dependent metabolization was determined using the fluorometric probe BFC as previously described^72^. Metabolization was assessed for 1 h and 4 h incubation times. For PHHs and data points that showed high values, samples were diluted 1/10 in buffer. The albumin secretion rate was quantified using the human albumin ELISA quantification kit (Bethyl) according to instructions provided in the kit.

### Drug biotransformation assessment

Comparative CYP450 biotransformation studies were performed to assess functionality across a number of gold standard primary hepatocyte models. Cryopreserved PHHs were obtained from a single donor (Lonza, Breda, The Netherlands) or pooled donors (Thermo Scientific, Merelbeke, Belgium). Upon thawing, cell suspensions (0.5 million cells/ml) were prepared in WE medium using 24-well plates (0.5 ml/well) on an orbital shaker and metabolization activity was assessed following a 15 min equilibration period. Human Hepatopac® plates were obtained from Ascendance Biotechnology (Ascendance Biotechnology, Boston USA) and cultures were maintained following instructions of the provider. Spheroid cultures were generated in-house as essentially described by Bell et al.^42^.

To assess the respective metabolization capacities, models were treated with a cocktail consisting of the probe substrates midazolam (10 µM), dextromethorphan (20 µM), phenacetin (100 µM), and tolbutamide (100 µM), to determine the activities of CYP3A4, CYP2D6, CYP1A2, and CYP2C9, respectively. Metabolite formation was quantified on a Shimadzu Nexera SIL-30AC system (Shimadzu Corp., Kyoto, Japan) connected to a SCIEX QTRAP 6500 mass spectrometer (SCIEX, Ontario, Canada) (for determining 4-OH-Tolbutamide and Phenacetin) or a SCIEX API-4000 (SCIEX) (for determining 1-OH-midazolam and dextrorphan) equipped with an ionization source operated in negative (4-OH-tolbutamide) or positive (1-OH-midazolam, dextrorphan, APAP) mode. Separation was carried out at 50 °C using either a Waters Acquity C18 BEH 1.7 µm (50 × 2.1 mm ID; Waters Corp., MA, USA) for midazolam and dextromethorphan or a Waters Acquity HSS T3 1.8 µm (2.1 × 100 mm; Waters Corp., MA, USA) for phenacetin or tolbutamide.

For quantification of 1-OH-midazolam and dextrorphan, a mobile phase was established consisting of solvent A (HPLC-grade water containing 0.1% formic acid) and solvent B (ACN). After a 2.5 min plateau at 60% (A)/40% (B), a solvent gradient was started from 60% (A)/40% (B) to 2% (A)/98%(B) over 0.5 min at a flow rate of 0.6 ml/min to elute the compounds from the column. Finally, the system was re-equilibrated at 60% (A)/40% (B) for an additional 0.7 min. Total run time was 3.8 min and 2 µl aliquots were injected onto the system for analysis.

For quantification of Phenacetin and 1-OH-tolbutamide, a mobile phase was established consisting of solvent A (HPLC-grade water containing 0.1% (vol:vol) acetic acid) and solvent B (methanol). After a 2 min plateau at 10% (A)/90% (B), a solvent gradient was initiated from 10% (A)/90% (B) to 2% (A)/98%(B) over 1 min at a flow rate of 0.4 ml/min to elute the compounds from the column, before the system was re-equilibrated at 10% (A)/90% (B) for an additional 1.0 min. Total run time was 5.0 min and 2 µl aliquots were injected into the system for analysis.

All data collection, processing, and analysis was performed with Analyst® software (v1.6.2. SCIEX). The lower limit of quantification was set at 0.5 ng/ml, 2.0 ng/ml, 0.1 ng/ml, 0.1 ng/ml, and 0.5 ng/ml for APAP, 4-OH-tolbutamide, dextrorphan, and 1-OH-midazolam, respectively.

### Western blot analysis

Cells were collected and lysed in RIPA lysis (Sigma-Aldrich) and extraction buffer (Thermo Scientific) supplemented with proteinase (complete, Mini Protease Inhibitor Cocktail Tablets provided in a glass vial, Roche, 11836153001) and phosphatase (PhosSTOP^**™**^, Sigma, 4906845001) inhibitors. Protein amount was measured using a pierce BCA protein assay kit (Thermo Scientific). Aliquots of 25 µg of protein were loaded on a NuPAGE 4–12% denaturing Bis-Tris gel and transferred to a nitrocellulose membrane (Thermo Scientific). Membranes were incubated overnight at 4 °C with primary antibodies and afterwards incubated with horseradish peroxidase-linked mouse secondary antibodies (Cell Signaling Technology #7076), and bound antibodies visualized using Pierce ECL reagent (Thermo Scientific).

### Enzymatic activity of mitochondrial proteins

The activity of citrate synthase and the individual complexes of the ETC (complex I to complex IV) in cell lines was determined as described before^73^. For HepG2 and HC3X-derived hepatocyte, pellets from five T-150 flasks were pooled as one sample. Snap-frozen pellets were homogenized using a glass homogenizer and motor-driven teflon plunger in homogenization buffer containing HEPES, EGTA, and sucrose. Enriched mitochondrial fractions were prepared by differential centrifugation at 4 °C (600 × *g* for 10 min to obtain crude mitochondrial fraction in the supernatant and subsequent 144,000 × *g* for 10 min for the enriched fraction in the resulting pellet). All pellets, except for those used for measuring complex III activity were resuspended in hypotonic buffer containing potassium phosphate and MgCl_2_. Citrate synthase and complex I–IV activities were determined by monitoring appearance or disappearance of specific substrates at specific wavelengths using spectrophotometry (e.g., oxidation of NADH for complex I activity at 340 nm in the presence and absence of rotenone to evaluate complex I-specific activity, reduction of cytochrome c for complex III activity at 550 nm).

### Toxicity measurements upon drug administration

PSCs were differentiated in a 384-well format. Cells were treated with 14 different concentrations of compounds in 6 repeats. As a negative control, 41 wells were left untreated. HepG2 were seeded at 40,000 cells per well. For control conditions, HepG2 were exposed after 2 days of culture. For AAGLY conditions, HepG2 were maintained in the supplemented medium for 30 days before exposure. For single exposure of APAP, amiodarone, and rotenone, medium was aspirated after 24 h of exposure and fresh medium was added to the cells supplemented with 0.3 mM of DRAQ7 (Abcam, ab109202) and Hoechst dye (1/1000, Sigma, 33258) for 25 min at 37 °C. Cells were fixed with 4% PFA. Imaging was done using an Operetta High-Content Imaging System (Perkin Elmer) and image analysis was performed using Harmony software (Perkin Elmer). Relative cell number was calculated as the percentage of living cells (DRAQ7-positive amount subtracted from the Hoechst-positive amount) relative to the average of living cells in the control wells. As a positive control, 1 donor of PHHs was seeded at 10,000 cells per well. Drugs were administrated after 8 h of attachment. For repeated exposures of the 13 training compounds, amiodarone, nefazodone, paracetamol, tolcapone, diclofenac, ximelagatran, troglitazone, perhexiline, bosentan, entacapone, metformin, and pioglitazone, measurement of resazurin conversion was done as described by Sison-Young et al.^43^.

### Statistics

Comparisons between two data groups (with *n* ≥ 3 independent experiments) were analyzed using an unpaired or paired two-tailed Student’s *t*-test. Values of <0.05 were considered significant and are indicated in the graphs as *p < 0.05, **p < 0.01, or ***p < 0.001. All data represent the mean ± SEM (*n* ≥ 3).

### Reporting summary

Further information on research design is available in the Nature Research Reporting Summary linked to this article.

## Supplementary information

Supplementary Information

Reporting Summary

## Data Availability

The authors declare that all data supporting the findings of this study are available within the article, the Supplementary Information, or from one of the corresponding authors upon request. The RNAseq data that support the findings of this study (Figs. 5 and 6) have been deposited in the Gene Expression Omnibus (GEO) repository under accession number GSE140520. The queried microarrays (Fig. 1) were already available in ArrayExpress under accession number E-MTAB-3994. The source data for all main and Supplementary Figures are provided as a source data file.

## Source Data

Source Data

## Code availability

The codes used for analysis of the data are available from the corresponding authors upon reasonable request.
