# Supplementary material for: Amino acid levels determine metabolism and CYP450 function of hepatocytes and hepatoma cell lines
Source: Nat Commun. 2020 Mar 13;11:1393. doi: 10.1038/s41467-020-15058-6 (PMC7069944; doi:10.1038/s41467-020-15058-6)
Supplement: Supplementary file 1 — Supplementary Information [file 41467_2020_15058_MOESM1_ESM.pdf]

Supplementary Information

Amino acid levels determine metabolism and CYP450 function of hepatocytes and hepatoma cell lines

Boon *et al.*

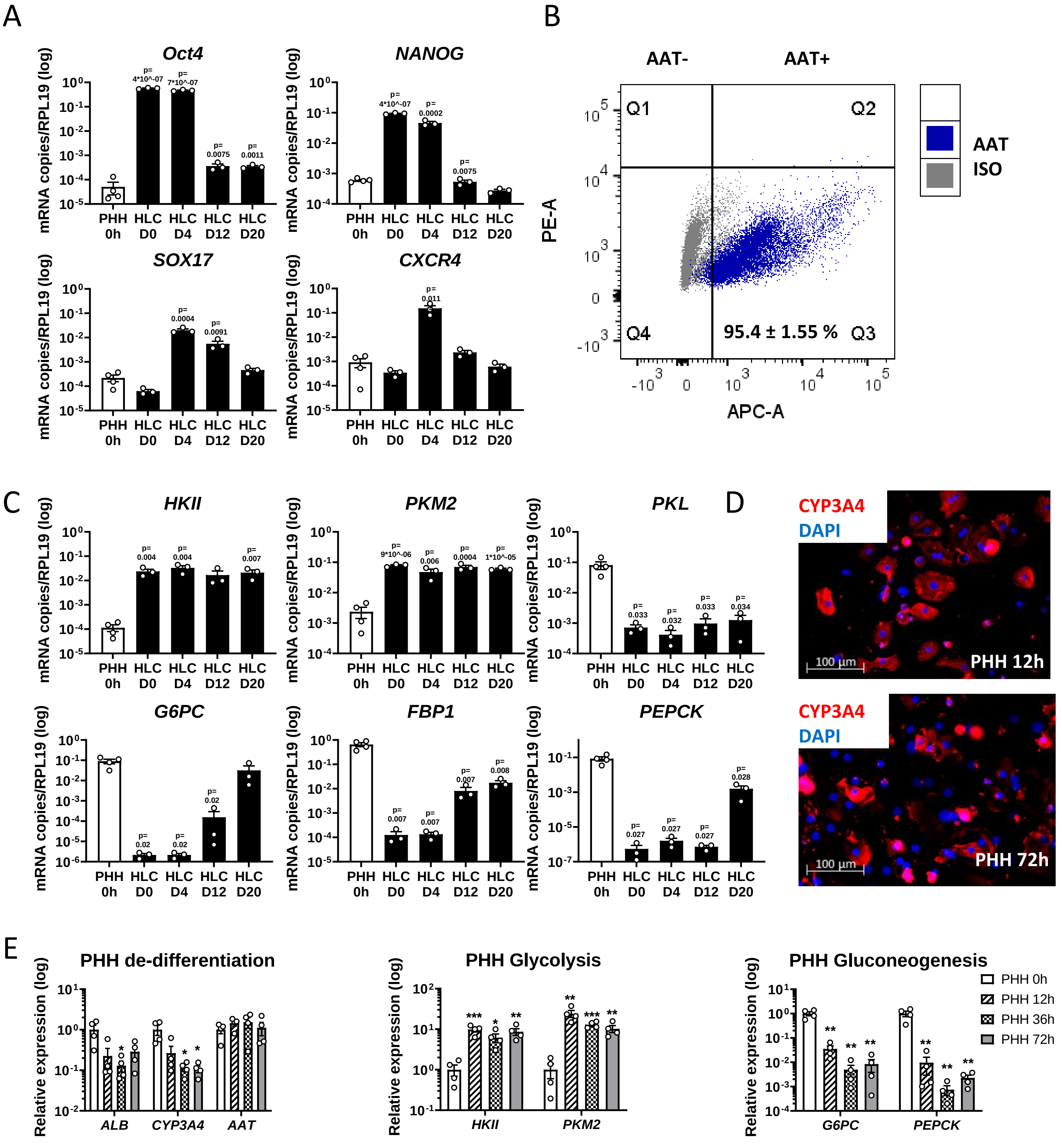

Supplementary figure 1. HLC D20 display a phenotype similar to that of 2D cultured PHHs. (A) Expression of *OCT4, NANOG, SOX17 and CXCR4 in* PHHs and HLCs at different time points (D0, D4, D12, D20). Data was normalized for expression of ribosomal protein lysine 19 (*RPL19*). N=3 independent differentiations. N=4 donors for PHHs. Significance was calculated as compared to PHH 0h by unpaired two-tailed Student's t-test. **(B)** Representative FACS plots for AAT, overlying isotype (gray) and AAT staining (blue) Quantification. N=4 independent differentiations. **(c)** Expression of *HKII, PKM2, PKL, G6PC, FBP1* and *PEPCK* in PHHs and differentiating HLCs at different time points (D0, D4, D12, D20). N=3 independent differentiations. N=4 donors for PHHs. Significance is calculated as compared to PHH 0h by unpaired two-tailed Student's t-test. **(D)** Immunostaining for CYP3A4 in PHH 12h and PHH 72h. representative image of plating of 2 donors of PHHs. (**E)** Relative expression of *ALB, CYP3A4*, AAT, HKII, PKM2, G6PC and PEPCK in PHH 12h, -36h, and -72h compared to PHH 0h. N=3 independent differentiations. N=4 donors for PHHs. Significance is calculated as compared to PHH 0h by unpaired two-tailed Student's t-test. ∗p < 0.05, ∗∗p < 0.01, ∗∗∗p < 0.001. Data in all panels represents mean ± standard error of mean (SEM) with P-values indicated when significant. Source data are provided as a Source Data file.

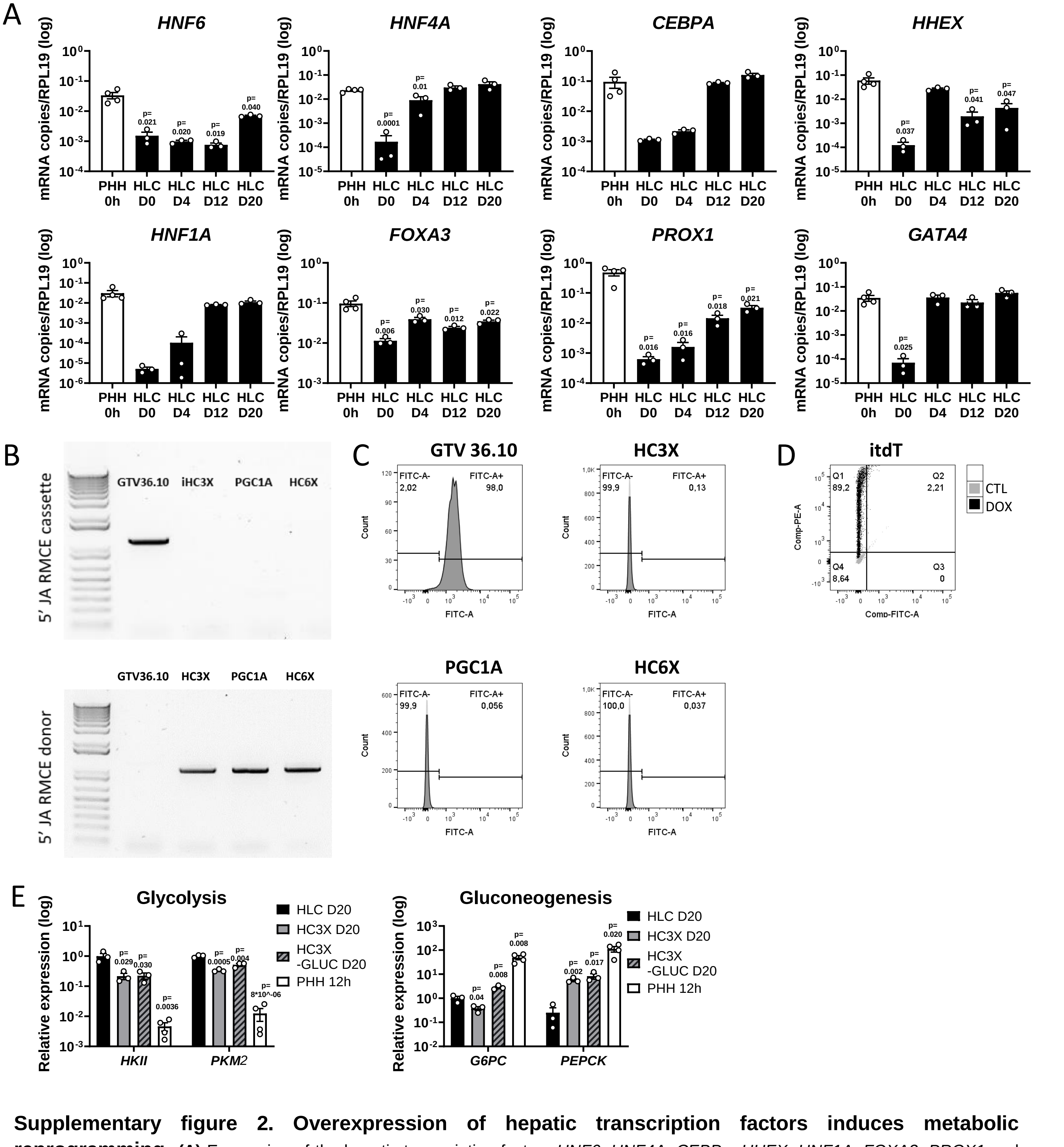

Supplementary figure 2. Overexpression of hepatic transcription factors induces metabolic reprogramming. **(A)** Expression of the hepatic transcription factors *HNF6, HNF4A, CEBPa, HHEX, HNF1A, FOXA3, PROX1* and *GATA4* in PHH 0h and HLCs at different time points (D0, D4, D12, D20). Data was normalized for expression of ribosomal protein lysine 19 (*RPL19*). N=3 independent differentiations. N=4 donors for PHHs. Significance is calculated as compared to PHH 0h by unpaired two-tailed Student's t-test. **(B)** PCR genotyping for presence of the exchangeable RMCE cassette (5'JA RMCE cassette) and of the integrated overexpression cassettes (5'JA RMCE donor) for the basic RMCE H9 cell line (GTV36,10) and the generated RMCE lines (HC3X, PGC1A and HC6X). One repeat. **(C)** FACS analysis demonstrating that following RMCE, the GFP cassette in the initial FRT-GFP.HYG/TK-FRT line in the *AAVS1* locus was eliminated: GFP signal for GTV36.10, HC3X, PGC1A and HC6X. One repeat. **(D)** FACS analysis for tdT signal in HLC D20 following RMCE with a tdT cassette and addition of 5µg/ml doxycycline from D4 to D20 (DOX) compared with HLC D20-tdT without doxycycline administration. Representative plot for 3 repeats. **(E)** Relative expression of genes involved in glycolysis and gluconeogenesis In HLC D20 and HC3X D20 when compared to PHH 0h. Data was normalized for expression of ribosomal protein lysine 19 (*RPL19*). N=3 independent differentiations. N=3 donors for PHHs. Significance is calculated as compared to HLC D20 by unpaired two-tailed Student's t-test. Data in all panels represents mean ± standard error of mean (SEM) with P-values indicated when significant. Source data are provided as a Source Data file.

Amino Acid	LDM (mg/l)	AA1 (mg/l)	AA2 (mg/l)	AA3 (mg/l)	AA3+GLY mg/l)	William's E (mg/l)
Glycine	3.01	17.96	62.77656	122.53512	20122.53	50
L-Alanine	3.56	21.29	74.47888	145.39376	145.39	90
L-Asparagine	60	85.2	160.8	261.6	261.6	20
L-Aspartic acid	5.2	31.69	111.184	217.168	217.16	30
L-Glutamic Acid	5.6	34.88	122.752	239.904	239.90	45
L-Proline	2.28	25.23	94.09	185.91	185.91	30
L-Serine	12.42	33.17	95.43	178.43	178.43	10
L-Arginine hydrochloride	25.25	25.25	88.19	277.04	277.04	50
L-Cystine	14	25.86	61.44	108.88	108.88	40
L-Histidine hydrochloride-H2O	8.02	28.94	91.70	175.38	175.38	15
L-Isoleucine	5.26	31.41	109.85	214.44	214.44	50
L-Leucine	15.66	41.70	119.83	224	224	75
L-Lysine hydrochloride	15.27	51.37	159.66	304.05	304.05	87
L-Methionine	1.76	9.29	31.88	62.01	62.01	15
L-Phenylalanine	1.96	18.44	67.88	133.80	133.80	25
L-Threonine	14.05	37.71	108.69	203.33	203.33	40
L-Tryptophan	2.40	7.48	22.71	43.01	43.01	10
L-Tyrosine	4.47	22.42	76.29	148.11	148.11	50
L-Valine	14.05	37.31	107.09	200.131888	200.13	50
L-Glutamine	408.86	404.7714	392.5056	376.1512	376.1512	292
Total Amount	623.08	991.3714	2159.227	3821.274	23821.25	1074

Supplementary table 1. Concentrations of AAs in utilized media and in William's E medium. Table showing the amino acid composition of utilized media.

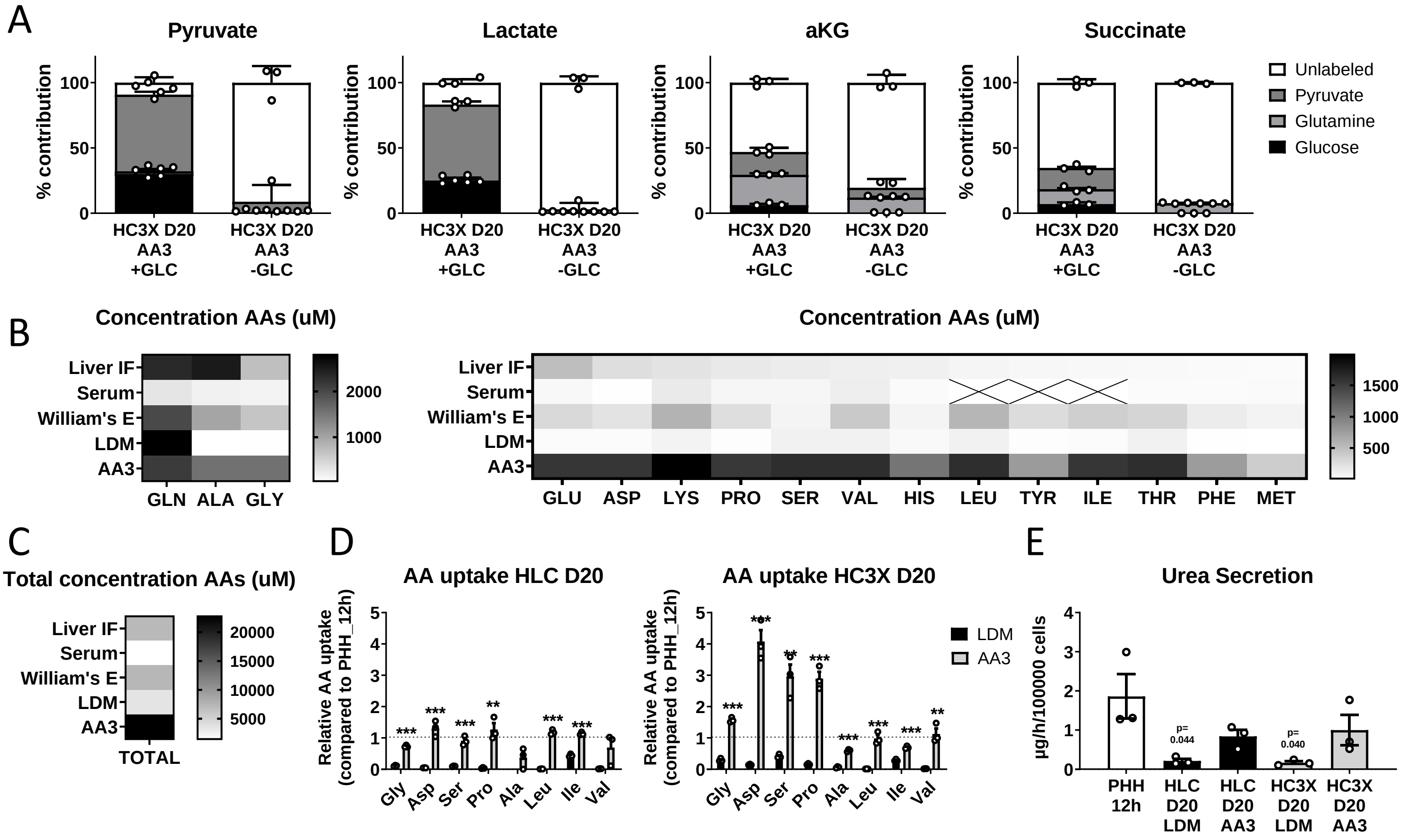

Supplementary figure 3. AA supplementation induces metabolic reprogramming. **(A)** Percentage of ^{13}C labeled glucose, glutamine or pyruvate contribution to pyruvate, lactate, alpha-ketoglutarate (AKG) and succinate in HC3X D20 cultured in medium supplemented with AA3 supplement with glucose (AA3+GLLC) and AA3 supplement without glucose (AA3-GLC). The non-labeled fraction was designated as unlabeled. N=3 independent differentiations. **(B)** and **(C)** Concentration of AAs found in mouse liver interstitial fluid, mouse serum, and in the different media used. Intensity of color correlates with concentrations. X signifies undetectable values. N= 5 mice for IF and N= 13 mice for serum measurements. **(D)** Amino acid uptake rates for glycine (GLY), aspartate (ASP), serine (SER), proline (PRO), alanine (ALA), leucine (LEU), isoleucine (iLEU) and valine (VAL) in HLC D20 and HC3X D20 cultured in LDM medium and LDM medium with AA3 supplementation (AA3), compared to PHH 12h cultured in WE medium (stippled line). N=3 independent differentiations, N=3 donors for PHHs. Significance is calculated by unpaired two-tailed Student's t-test by comparing against PHH 12h.$**p < 0.01$, $***p < 0.001$. **(E)** Urea secretion rates for HLC and HC3X D20 progeny cultured in LDM medium and LDM medium with AA3 supplementation (AA3) compared to PHH 12h cultured in William's E medium. N=3 independent differentiations, N=3 donors for PHHs. Significance is calculated by unpaired two-tailed Student's t-test compared with PHH 12h. Data in all panels represents mean ± standard error of mean (SEM) with P-values indicated when significant. Source data are provided as a Source Data file.

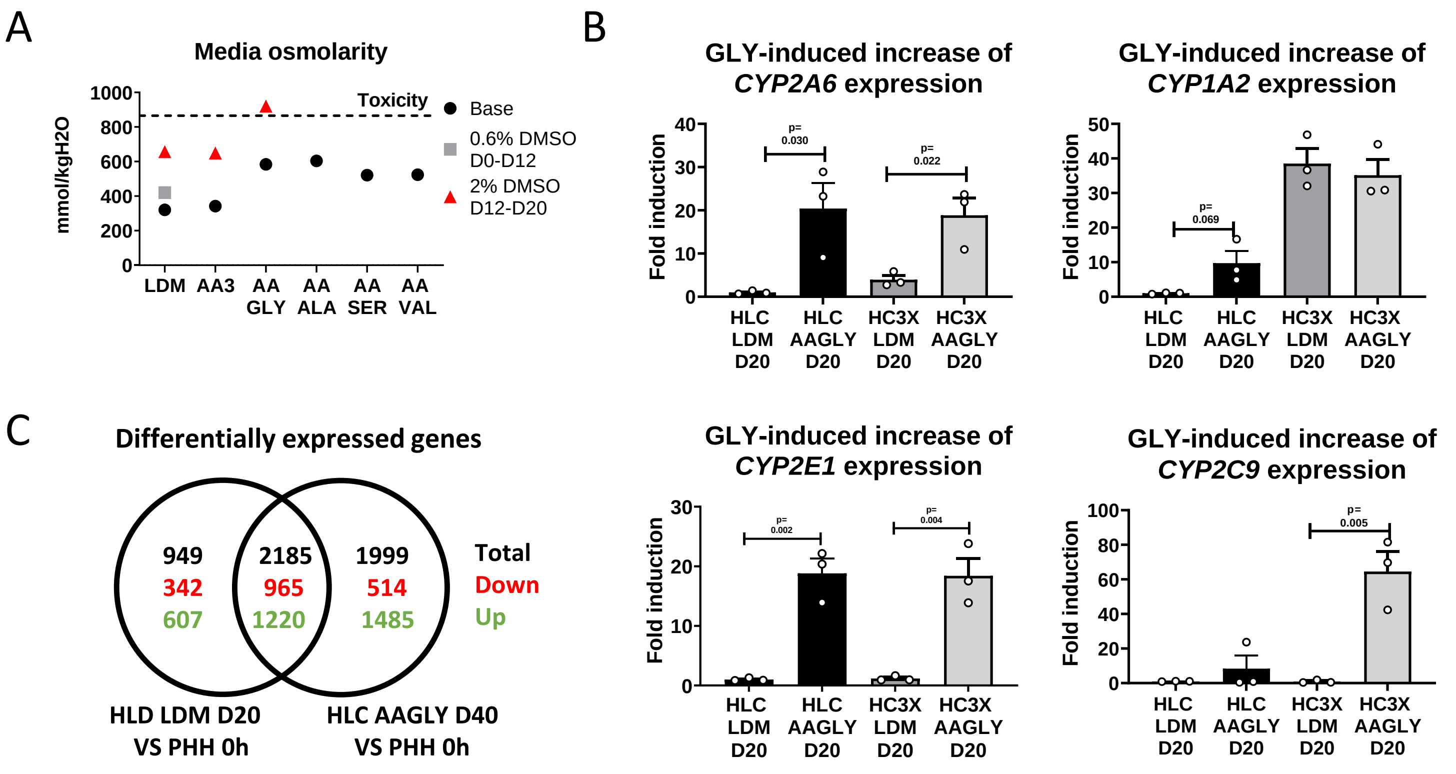

Supplementary figure 4. Elevating AA levels induces CYP450 function and global maturation of HLC and HC3X. **(A)** Osmolarity of different media compositions. **(B)** Relative expression of *CYP450* isoforms in HLCs and HC3X-progeny cultured with or without AAGLY-supplementation from D14 until D20 of differentiation. N=3 independent differentiations. Significance is compared to LDM conditions and calculated by two-tailed unpaired Student;s t-test. **(C)** Venn diagram displaying the number of up- and downregulated genes when comparing PHHs to either HLC LDM D20 or HLC AAGLY D40. N=3 independent differentiations Data in all panels represents mean ± standard error of mean (SEM) with P-values indicated when significant. Source data are provided as a Source Data file.

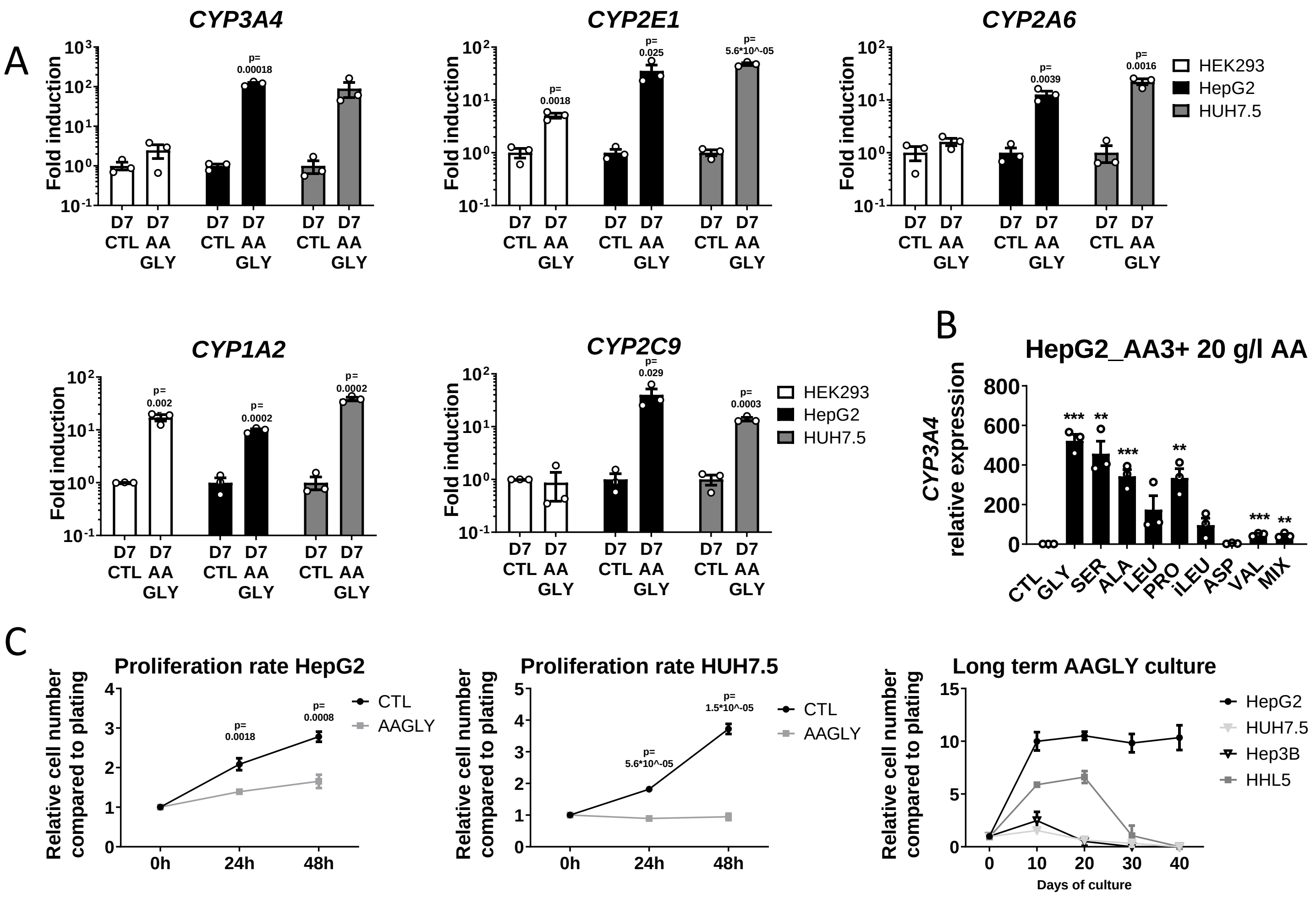

Supplementary figure 5. AA supplementation drives global differentiation of HepG2. **(A)** Relative expression of *CYP450* isoforms in HepG2 cultured with or without AAGLY-supplementation for 7 days. N=3 independent maturations. Significance is compared to CTL media by unpaired two-tailed Student's t-test. **(B)** Relative gene expression analysis for *CYP3A4* in HepG2 cultured in medium supplemented with AA3 with or without 2% of either glycine (GLY), serine (SER), alanine (ALA), leucine (LEU), proline (PRO), isoleucine (iLEU), aspartate (ASP), valine (VAL), or a mix of all of the above. N=3 independent maturations. Significance is calculated compared to CTL medium by unpaired two-tailed Student's t-test. $**p < 0.01$, $***p < 0.001$. **(C)** Relative proliferation rates for HepG2, HUH7.5, HHL5 and Hep3B cells grown with or without AAGLY-Supplementation. N=3 independent treatments. Significance is compared to CTL conditions by upaired two-tailed Student's t-test. Data in all panels represents mean ± standard error of mean (SEM) with P-values indicated when significant. Source data are provided as a Source Data file.

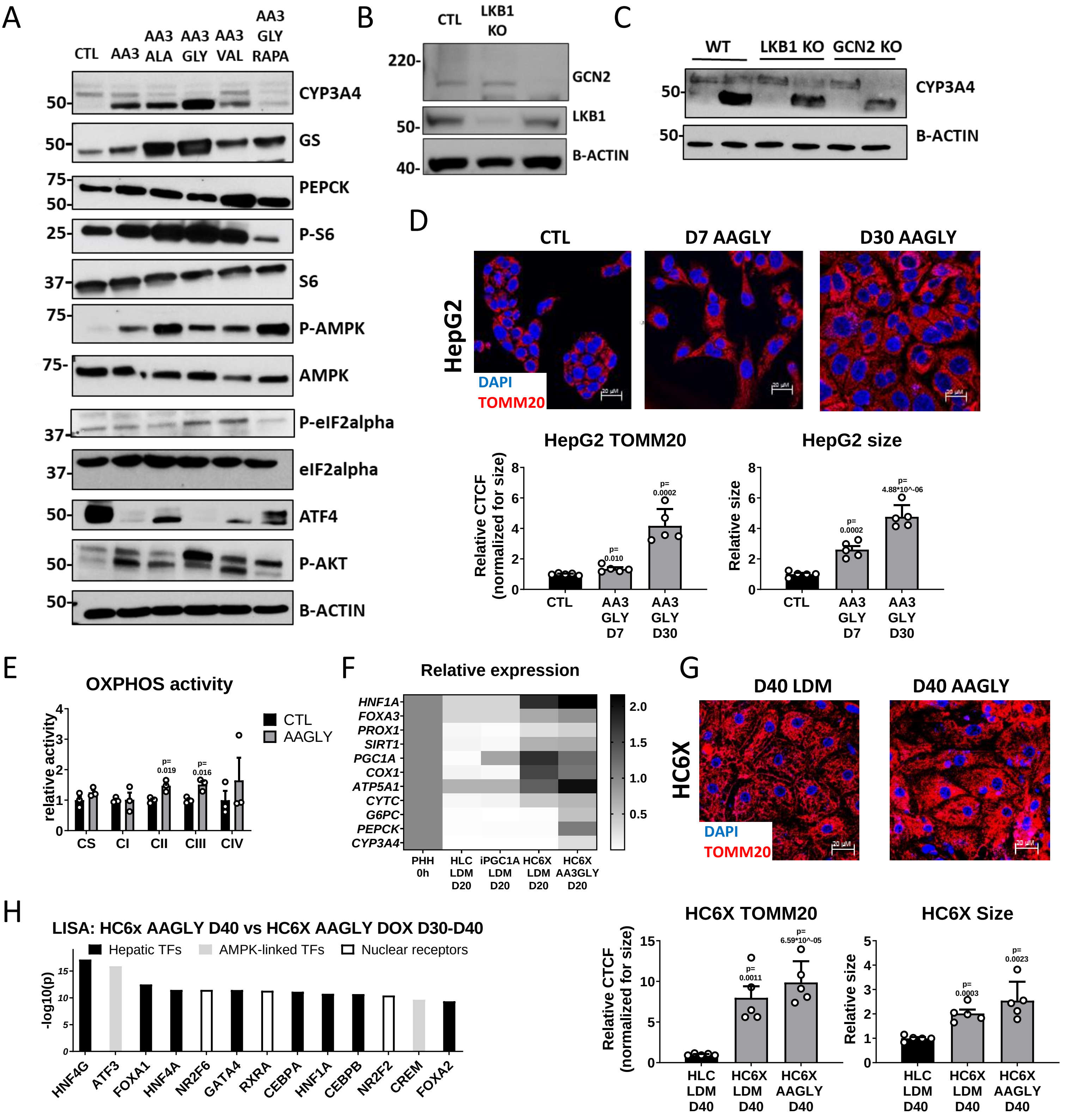

Supplementary figure 6. AAs allow activation of the hepatic transcriptional network **(A)** Western blot showing expression of hepatic markers and the activity of metabolic signaling pathways in HepG2 D30 differentiated in standard media (CTL), in AA3+ALA, AA3+GLY or AA3+VAL medium, or in AA3+GLY media supplemented with rapamycin (RAPA). N=2. **(B)** Western blot analysis for GCN2 or LKB1 in WT or KO HepG2. N=1**(C)** Western blot showing induction of CYP3A4 in control or KO HepG2 after AAGLY-mediated differentiation at day 30. N=1 **(D)** Staining for the mitochondrial subunit TOMM20 and analysis of cell size and normalized TOMM20 intensity of HepG2 grown with or without AAGLY. Representative pictures (N=mean of 10 random images of 5 wells). Significance is calculated by comparing to CTL by unpaired two tailed Student's t-test. **(E)** Activity measurements of the different mitochondrial complexes (CI, CII, CIII, and CIV) and for the mitochondrial enzyme citrate synthase (CS) in HepG2 grown with or without AAGLY supplementation for 7 days. N=3 independent differentiations. Significance is calculated by comparing to CTL by paired two tailed Student's t-test. $*p < 0.05$ **(F)** Heatmap representing mean expression levels for transgenes, and genes linked to the PGC-1α pathway in PHHs, HLCs and iPGC1A or HC6X cells. N=3 independent differentiations. **(G)** Staining for the mitochondrial subunit TOMM20 and analysis of cell size and normalized TOMM20 intensity for HC6X D20 grown with or without AAGLY supplementation. Representative pictures. N=mean of 10 random images from 5 differentiations. Significance is calculated by comparing to CTL by unpaired two tailed Student's t-test. **(H)** LISA analysis identifying top TFs responsible for the difference between HC6X AAGLY D40 and HC6X AAGLY D40 with removal of doxycycline between D30 and D40. Data in all panels represents mean ± standard error of mean (SEM) with P-values indicated when significant. Source data are provided as a Source Data file.

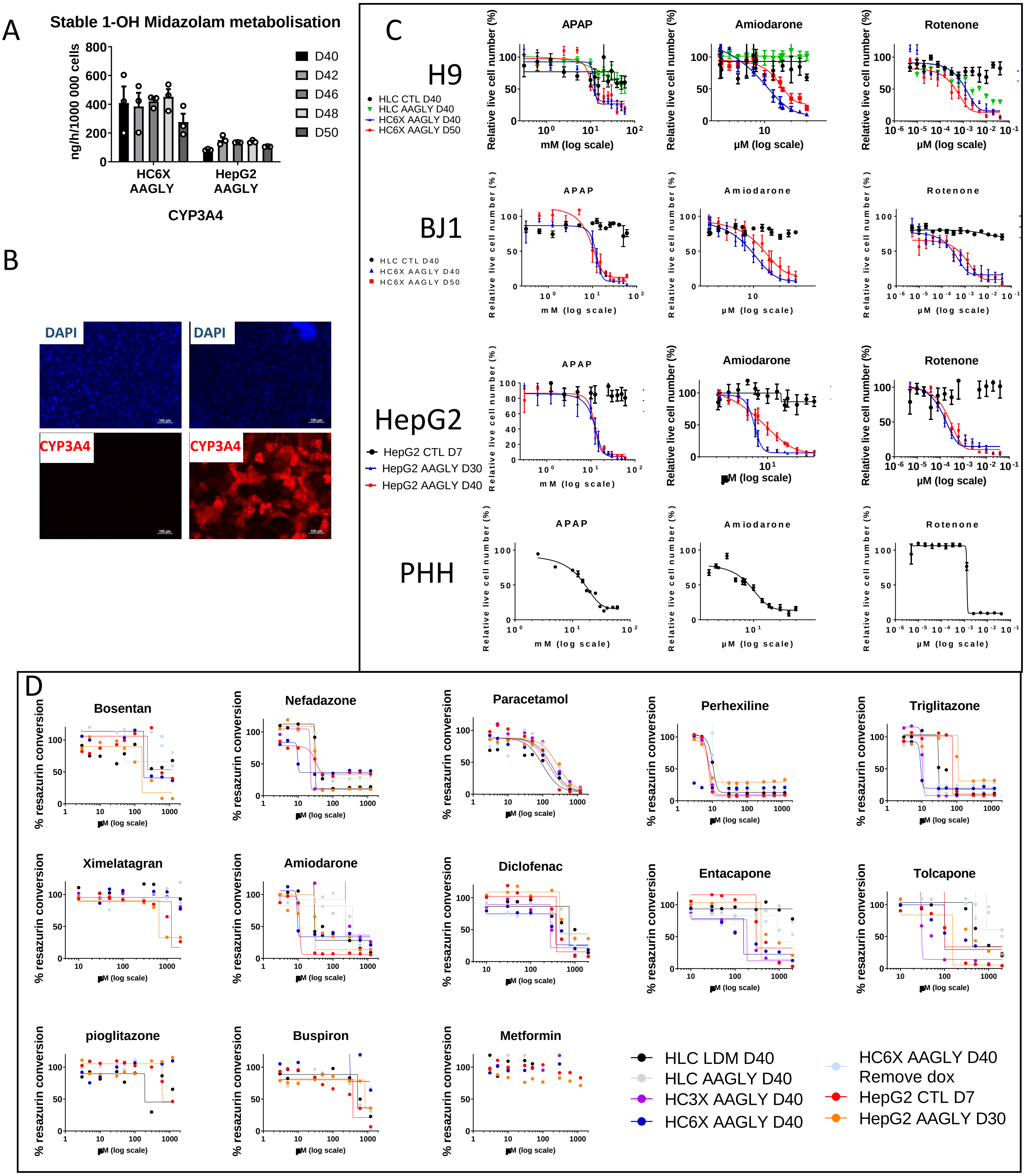

Supplementary figure 7 AA supplementation allows for identifying DILI compounds. **(A)** CYP3A4-dependent metabolization of 1-OH midazolam over a 10-day period in HC6X AAGLY D40 and HepG2 AAGLY D30 (N=1 treatment of 3 simultaneous differentiation). **(B)** Representative image of CYP3A4 staining in HC6X LDM D40 and HC6X AAGLY D40 cells for two independent differentiations **(C)** Relative number of living cells measured by Hoechst and Draq7 staining after 24h exposure to different concentrations of acetaminophen (APAP), rotenone and amiodarone. As a positive control PHH 12h (2 donors) grown in WE medium were used. Sigmoidal kill curves were plotted using Graphpad Prism software. N=6 wells per concentration for treated cells. N= 18 wells for control cells. 6 replicate samples of 1 donor of PHHs were analyzed. **(D)** Sigmoidal kill curves representing resazurin conversion upon 3 day exposure to 9 hepatotoxic and 4 non-hepatotoxic compounds. N=6 wells for treated conditions, N= 14 wells for control conditions. Data in all panels represents mean ± standard error of mean (SEM). Source data are provided as a Source Data file.

Drug	Reference	Cell model	Exposure time	Measurement	IC50 (µM)
APAP (Boon et al: 10 000 µM)	[10]	Spheroid of immortalized PHHs	24 h	ATP	40 000
	[3]	Spheroid of PHHs	48h	ATP	10 000
	[11]	3D Bioreactor with primary rat hepatocytes	24h	Live Death	14 290
	[12]	Co-culture of endothelial cells and mouse hepatocytes	36h	Cell count	7000
	[13]	Primary rat hepatocytes	24h	WST-1 assay	14 000
	[14]	Rat liverbeads (Alginate beads of rat hepatocytes)	24h	LDH	10 240
	[15]	PHH	24h	ALT release	± 10 000
	[16]	Cryopreserved PHH	48h	ATP	> 25 000
	[17]	Rat hepatocytes	24h	MTT	7 600
		Human cryopreserved hepatocytes	24h		28 200
	[4]	Co-culture of PHHs and fibroblasts	24h	ATP	5 500
Amiodarone (Boon et al. 5-10 µM)	[10]	Spheroid of immortalized PHHs	24 h	ATP	260
	[3]	Spheroid of PHHs	48h	ATP	>100
	[4]	Co-culture of PHHs and fibroblasts	24h	ATP	33

Supplementary table 2. IC50 values for APAP and amiodarone exposure to PHH systems in published studies.

Reference	Cell model	Basal media	Total AA concentration
[1]	Primary hepatocytes	Williams' Medium E	0.782 g/l
[2]	Primary hepatocytes	William's E medium	0.782 g/l
[3]	Primary hepatocytes	William's E medium with 2 mM of glutamine	1.075 g/l
[4]	Primary hepatocytes	William's E medium with 2 mM of glutamine	1.075 g/l
[5]	PSC differentiation	CMRL/Hepatozyme (Invitrogen)	0.944 g/l
[6]	PSC differentiation	Hepatocyte Basal Medium (Lonza CC-3199)	0.782 g/l
[7]	PSC differentiation	Williams' E medium	0.782 g/l
[8]	PSC differentiation	DMEM/F12 with 1 mmol/L nonessential amino acids	1.189 g/l
[9]	PSC differentiation	RPMI B27	0.86808 g/l
Boon et al	PSC differentiation Cell line maturation	AA3-medium	3.696 g/l
Boon et al	PSC differentiation Cell line maturation	AA3-glycine medium	23.696 g/l

Supplementary table 3 Comparison of AA concentrations in PHH culture and HLC differentiation. Comparison of the total amount of amino acids present in hepatic differentiation and maintenance systems.

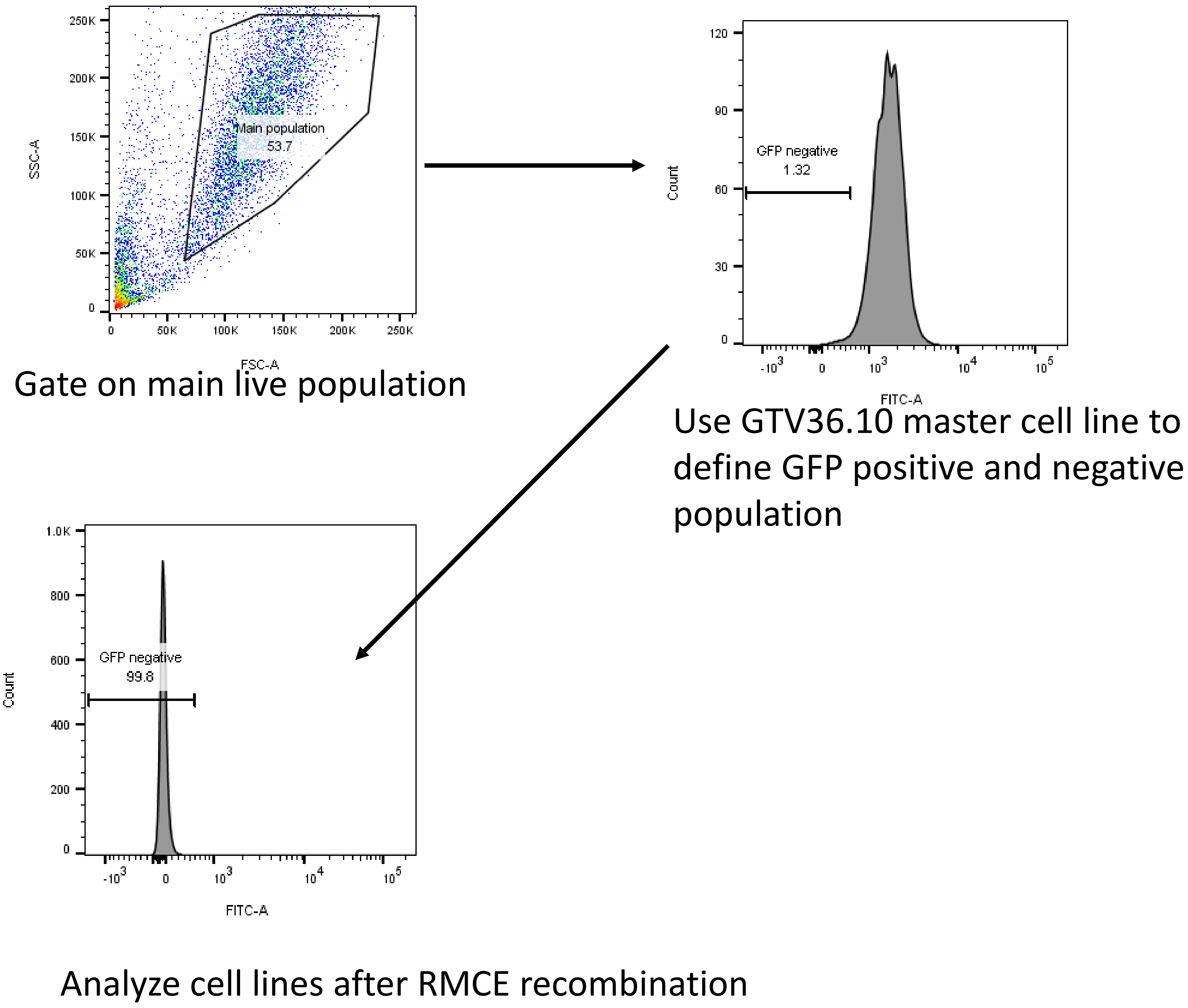

Supplementary figure 8. Flow cytometry gating strategy for GFP signal utilized in supplementary Figure 2C. This representative schema describes the gating for evaluating RMCE exchange efficiency for the generation of new RMCE PSC lines. Exchange of the master RMCE cassette was evaluated by loss of GFP signal. In order to evaluate % of GFP positivity, we first gated on the main population in a FSC-A/ SSC-A plot as shown in A. During this step we excluded the FSC-A low/ SSC-low population. The same gating was used for all samples. Next, we separated negative and positive cells based on signal from the GFP positive RMCE master cell line GTV36.10 as as shown in B. We then analyzed the loss of GFP in exchanged cell lines as shown in C.

Liver Differentiation Media (LDM)	Catalog number	Supplier	Volume for 500 ml (ml)
DMEM LG	31885	Invitrogen	285
MCDB pH=7.2	M6770	Sigma	200
Pen/Strep	15140	Invitrogen	5
L-Ascorbic Acid	A8960	Sigma	5
ITS	4140-045	Invitrogen	1.25
LA-BSA	L9530	Sigma	1.25
b-Mercapto	31350	Invitrogen	0.5
Dexamethasone	D-2915	Sigma	2

HepG2,Hep3B and HHL5	Catalog number	Supplier	Volume for 500 ml (ml)
DMEM LG	31885	Invitrogen	445
FBS	F7524	Sigma	50
Pen/Strep	15140	Invitrogen	5

Huh7.5	Catalog number	Supplier	Volume for 500 ml (ml)
DMEM HG	41965	Invitrogen	440
FBS	F7524	Sigma	50
Pen/Strep	15140	Invitrogen	5
NEAA	11140	Invitrogen	5

HEK293T	Catalog number	Supplier	Volume for 500 ml (ml)
DMEM HG	41965	Invitrogen	429
FBS	F7524	Sigma	50
Pen/Strep	15140	Invitrogen	5
L-Glutamine	25030	Invitrogen	10
Sodium Pyruvate	11360	Invitrogen	6

Supplementary table 4. Media composition. Media compositions of all basal media used.

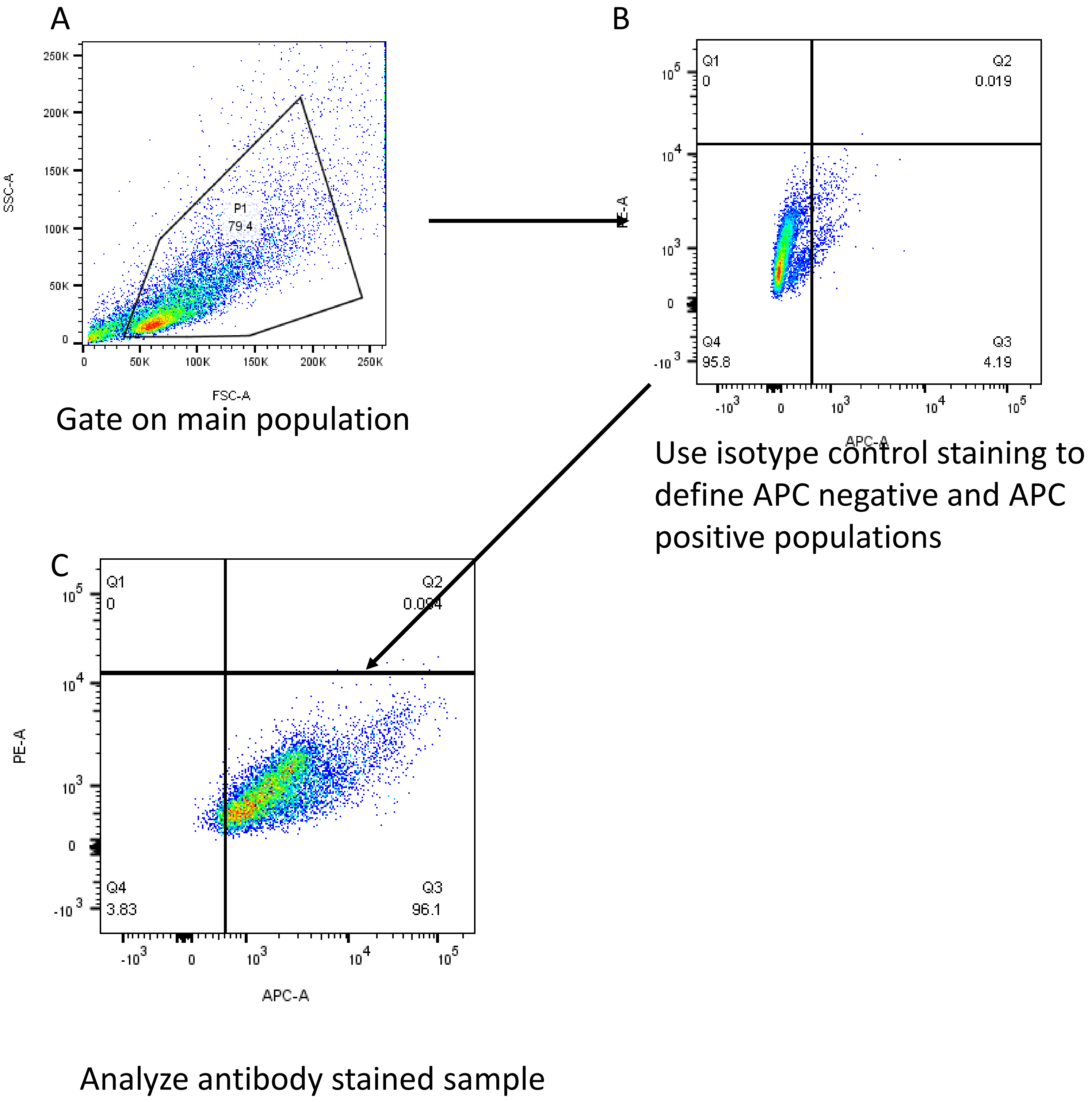

Supplementary figure 9. Flow cytometry gating strategy for AAT staining utilized in supplementary Figure 1B. This representative schema describes the gating for PSC-derived HLC cells stained with an antibody for AAT. Cells were fixed, permeabilized and stained according to the description in the material and methods. In order to evaluate % of AAT positivity, we first gated on the main population in a FSC-A/ SSC-A plot as shown in A. During this step we excluded the FSC-A low/ SSC-low population. The same gating was used for all samples. Next, we separated negative and positive cells based on an isotype staining as shown in B. We then analyzed the AAT-stained samples as shown in C.

Name	Company	CAT-NR (LOT-NR)	Dilution
Rabbit anti-Oct4 (H-134)	Santa Cruz	sc-9081	1:100
Mouse anti-HNF4α (K9218)	Abcam	ab41898 (72)	1/200
Rabbit anti-SLC10A1=NTCP	Sigma	HPA042727 (C106087)	1/500
Polyclonal Rabbit Anti-Human Alpha-1-Antitrypsin (Multipurpose)	DAKO	A0012 (00092029)	
Anti-Human CYP3A4	Tebu-Bio (Cypex limited (Dundee, UK))	PAP 011 (150616)	1/100
Rabbit anti-PEPCK (H-300)	Santa Cruz	sc-32879 (F2008)	1/1000
Donkey anti-Mouse IgG (H+L) Highly Cross-Adsorbed Secondary Antibody. Alexa Fluor 488	Molecular probes	A-11029	1/500
Donkey anti-Rabbit IgG (H+L) Highly Cross-Adsorbed Secondary Antibody. Alexa Fluor 555	Molecular probes	A21429	1/500
Rabbit IgG	BD Pharmingen	550875 (6175659)	Used as control in same concentration as primary antibody
Mouse BALB/c IgG1. κ	BD Pharmingen	550878	Used as control in same concentration as primary antibody
p70 S6 Kinase Antibody (49D7)	Cell signaling technology.	2708	1/1000
Phospho-p70 S6 Kinase (Thr389) Antibody (108D2)	Cell signaling technology.	9234 (12)	1/1000
S6 Ribosomal Protein (5G10) Rabbit mAb	Cell signaling technology.	2217 (7)	1/1000
Phospho-S6 Ribosomal Protein (Ser235/236) Antibody (D57.2.2E)	Cell signaling technology.	4858 (16)	1/1000
Anti-rabbit IgG. HRP-linked Antibody	Cell signaling technology.	7074	1/10000
Anti-TOMM20 antibody	Abcam	ab56783	1/500
Anti-B actin (D6A8)	Cell Signaling	8475	1/1000
Anti GCN2 (E9H60)	Cell Signaling	40457S	1/1000
Anti LKb1 (27D10)	Cell Signaling	3050S	1/1000
ANTI P- eIF2alpha (ser51)	Cell Signaling	9721 (21)	1/500
ANTI- eIF2alpha (D7D3)	Cell Signaling	5324 (6)	1/1000
Anti ATF4 (D4B8)	Cell Signaling	11815S	1/1000
Anti P-AMPK (T172)(40H9)	Cell Signaling	2535 (21)	1/1000
Anti-AMPK (D5A2)	Cell Signaling	5831 (6)	1/1000
Anti-Hamartin (TSC1)(5C8A12)	Thermo Fisher	37-0400 (QG21065)	1/2000

Supplementary table 5. Antibodies. Table showing all relevant antibodies used in this study

Primer name	Sequence	Primer name	Sequence
RPL19	ATT GGT CTC ATT GGG GTC TAA C<br>AGT ATG CTC AGG CTT CAG AAG A	HNF4A	ACTACGGTGCCTCGAGCTGT<br>GGCACTGGTTCCTCTTGTCT
AAT	AGGGCCTGAAGCTAGTGGAT<br>TCCTCGGTGTCCTTGACTTC	CEBPa	AAAGGGGTGGAAACATAGGG<br>GGAGAGGCGTGGAACTAGAG
NTCP	ATCGTCCTCAAATCCAAACG<br>CCACATTGATGGCAGAGAGA	HHEX	AGGAGAACCCTCAAAGCA<br>TCTGAACATGCCAATGCC
ALB	ATGCTGAGGCAAAGGATGTC<br>AGCAGCAGCACGACAGAGTA	GATA4	TCCAAACCAGAAAACGGAAG<br>CTGTGCCCGTAGTGAGATGA
OCT4	GATGGCGTACTGTGGGCCC<br>TGGGACTCCTCCGGGTTTTG	CYP2A6	GTTGTACATCTGCTCTGTGTTC<br>GTGGCCTTGCTGGTCTG
NANOG	CCTGTGATTTGTGGGCCTG<br>GACAGTCTCCGTGTGAGGCAT	CYP1A2	CAGCTCTGGGTCATGGTTG<br>CCTCCTTCTTGCCCTTCAC
SOX17	CGCTTTCATGGTGTGGGCTAAGGACG<br>TAGTTGGGGTGGTCCTGCATGTGCTG	CYP2E1	GCACACAACAAAAGAAACAACTC<br>AGCCAGAACACTTCCTGAATG
CXCR4	CACCGCATCTGGAGAACCA<br>GCCCATTTCCTCGGTGTAGTT	PGC1A	CCTTGCAGCACAAGAAAACA<br>TGCTTCGTCGTCAAAAACAG
HKII	CTTCTTCACGGAGCTCAACC<br>AGCCCTTTCTCCATCTCCTT	SIRT1	CATAGACACGCTGGAACAGG<br>CAAATCAGGCAAGATGCTGT
PKM2	GAGGCCTCCTTCAAGTGC<br>CCAGACTTGGTGAGGACGAT	KAT2A	CTGGAAAAGTTCCGAGTGGA<br>GCCCCATAGATCTCCTCCTC
PKL	ACTAAGCCGTGATCCCACTG<br>CGGTACCGAGACAGAAGCTG	NRF1	TGTGGGACAGCAAGCTATTG<br>GCAGACTCCAGGTCTTCCAG
G6PC	GTGTCCGTGATCGCAGACC<br>GACGAGGTTGAGCCAGTCTC	NRF2	GACGGGATATTCTCTTCTGTGC<br>ACTCTTTCCGTCGCTGA
FBP1	ACGTCCAGCTTCTTAACTTGA<br>AGCAGTCAAAGCCATCTCTTC	TFAM	GGGAAGGTCTGGAGCAGAG<br>TGGACAACTTGCCAAGACAG
PEPCK	AAGAAGTGCTTTGCTCTCAG<br>CCTTAAATGACCTTGTGCGT	COX1	CATCAATGTCTCCATACAATTCCT<br>CTGCAGCCCTTCAATGAGT
HNF1A	ACACCTCAACAAGGGCACTC<br>TGGTAGCTCATCACCTGTGG	CYTC	TGTGCCAGCGACTAAAAAGA<br>AGATTTGGCCCAGTCTTGTG
FOXA3	ATTCTCTCTGGCATGGGTTG<br>AAATTCCCCACACCCTAACC	ATP5A1	TCCAGAAATGCTTTGGGTTC<br>GCTCCAAGAATACGCTCTTCA
FOXA _exogenous	TCAGAACAGACACCAGCACC<br>GGCCCTGGTAGTAGACTCCA	5'JA RMCE cassette	CACTTTGAGCTCTACTGGCTTC<br>CATGTTAGAAGACTTCCTCTGC
PROX1	TCACCTTATTCGGGAAGTGC<br>GGAGCTGGGATAACGGGTA	5'JA RMCE donor	TTCACTGCATTCTAGTTGTGG<br>AAGGCAGCCTGGTAGACA
HNF6	AAATCACCATTTCCCAGCAG<br>ACTCCTCCTTCTTGCGTTCA		
CYP3A4	TTCCTCCCTGAAAGATTCAGC<br>GTTGAAGAAGTCCTCCTAAGCT		

Supplementary table 6. Primer sequences. Table showing primer sequences used for qRT-PCR analysis

Supplementary references

1. Shulman, M. & Nahmias, Y. Long-Term Culture and Coculture of Primary Rat and Human Hepatocytes BT - Epithelial Cell Culture Protocols: Second Edition. in (eds. Randell, S. H. & Fulcher, M. L.) 287–302 (Humana Press, 2013). doi:10.1007/978-1-62703-125-7_17
2. Levy, G. *et al.* Long-term culture and expansion of primary human hepatocytes. *Nat Biotech* **33**, 1264–1271 (2015).
3. Bell, C. C. *et al.* Transcriptional, functional and mechanistic comparisons of stem cell-derived hepatocytes, HepaRG cells and 3D human hepatocyte spheroids as predictive in vitro systems for drug-induced liver injury. *Drug Metab. Dispos.* (2017).
4. Novik, E. I. *et al.* Long-enduring primary hepatocyte-based co-cultures improve prediction of hepatotoxicity. *Toxicol. Appl. Pharmacol.* **336**, 20–30 (2017).
5. Rashid, S. T. *et al.* Modeling inherited metabolic disorders of the liver using human induced pluripotent stem cells. *J. Clin. Invest.* **120**, 3127–3136 (2010).
6. Hannan, N. R. F., Segeritz, C.-P., Touboul, T. & Vallier, L. Production of hepatocyte like cells from human pluripotent stem cells. *Nat. Protoc.* **8**, 430–437 (2013).
7. Avior, Y. *et al.* Microbial-derived lithocholic acid and vitamin K2 drive the metabolic maturation of pluripotent stem cells-derived and fetal hepatocytes. *Hepatology* **62**, 265–278 (2015).
8. Chen, Y. *et al.* Amelioration of Hyperbilirubinemia in Gunn Rats after Transplantation of Human Induced Pluripotent Stem Cell-Derived Hepatocytes. *Stem cell reports* **5**, 22–30 (2015).
9. Peters, D. T. *et al.* Asialoglycoprotein receptor 1 is a specific cell-surface marker for isolating hepatocytes derived from human pluripotent stem cells. *Development* **143**, 1475–1481 (2016).
10. Fey, S. J. & Wrzesinski, K. Determination of Drug Toxicity Using 3D Spheroids Constructed From an Immortal Human Hepatocyte Cell Line. *Toxicol. Sci.* **127**, 403–411 (2012).
11. Toh, Y.-C. *et al.* A microfluidic 3D hepatocyte chip for drug toxicity testing. *Lab Chip* **9**, 2026–2035 (2009).
12. Toyoda, Y. *et al.* Acetaminophen-induced hepatotoxicity in a liver tissue model consisting of primary hepatocytes assembling around an endothelial cell network. *Drug Metab. Dispos.* **40**, 169–177 (2012).
13. Wang, K., Shindoh, H., Inoue, T. & Horii, I. Advantages of in vitro cytotoxicity testing by using primary rat hepatocytes in comparison with established cell lines.Wang, K. et al., 2002. Advantages of in vitro cytotoxicity testing by using primary rat hepatocytes in comparison with established cell . *J. Toxicol. Sci.* **27**, 229–237 (2002).
14. Biagini, C. P. *et al.* Investigation of the hepatotoxicity profile of chemical entities using Liverbeads® and WIF-B9 in vitro models. *Toxicol. Vitr.* **20**, 1051–1059 (2006).
15. Xie, Y. *et al.* Mechanisms of acetaminophen-induced cell death in primary human hepatocytes. *Toxicol. Appl. Pharmacol.* **279**, 266–274 (2014).
16. Yokoyama, Y. *et al.* Comparison of drug metabolism and its related hepatotoxic effects in HepaRG, cryopreserved human hepatocytes, and HepG2 cell cultures. *Biol. Pharm. Bull.* (2018). doi:10.1248/bpb.b17-00913
17. Jemnitz, K., Veres, Z., Monostory, K., Kóbori, L. & Vereczkey, L. Interspecies differences in acetaminophen sensitivity of human, rat, and mouse primary hepatocytes. *Toxicol. Vitr.* **22**, 961–967 (2008).
